# Recent Discoveries of Macromolecule- and Cell-Based Biomarkers and Therapeutic Implications in Breast Cancer

**DOI:** 10.3390/ijms22020636

**Published:** 2021-01-10

**Authors:** Hsing-Ju Wu, Pei-Yi Chu

**Affiliations:** 1 Department of Biology, National Changhua University of Education, Changhua 500, Taiwan; hildawu09@gmail.com; 2 Research Assistant Center, Show Chwan Memorial Hospital, Changhua 500, Taiwan; 3 Department of Medical Research, Chang Bing Show Chwan Memorial Hospital, Lukang Town, Changhua County 505, Taiwan; 4 School of Medicine, College of Medicine, Fu Jen Catholic University, New Taipei City 231, Taiwan; 5 Department of Pathology, Show Chwan Memorial Hospital, No. 542, Sec. 1 Chung-Shan Rd., Changhua 500, Taiwan; 6 Department of Health Food, Chung Chou University of Science and Technology, Changhua 510, Taiwan; 7 National Institute of Cancer Research, National Health Research Institutes, Tainan 704, Taiwan

**Keywords:** breast cancer, biomarker, diagnosis, prognosis, treatment

## Abstract

Breast cancer is the most commonly diagnosed cancer type and the leading cause of cancer-related mortality in women worldwide. Breast cancer is fairly heterogeneous and reveals six molecular subtypes: luminal A, luminal B, HER2+, basal-like subtype (ER−, PR−, and HER2−), normal breast-like, and claudin-low. Breast cancer screening and early diagnosis play critical roles in improving therapeutic outcomes and prognosis. Mammography is currently the main commercially available detection method for breast cancer; however, it has numerous limitations. Therefore, reliable noninvasive diagnostic and prognostic biomarkers are required. Biomarkers used in cancer range from macromolecules, such as DNA, RNA, and proteins, to whole cells. Biomarkers for cancer risk, diagnosis, proliferation, metastasis, drug resistance, and prognosis have been identified in breast cancer. In addition, there is currently a greater demand for personalized or precise treatments; moreover, the identification of novel biomarkers to further the development of new drugs is urgently needed. In this review, we summarize and focus on the recent discoveries of promising macromolecules and cell-based biomarkers for the diagnosis and prognosis of breast cancer and provide implications for therapeutic strategies.

## 1. Introduction

Breast cancer is the most commonly diagnosed cancer type and the leading cause of cancer-related mortality in women worldwide [1]. It is estimated that there were approximately 2 million new cases and 627,000 breast cancer-related mortalities globally in 2018 [2,3]. Although the five-year relative survival rate for localized breast cancer is relatively high (80–92%), the survival rate dramatically declines to <25% for metastatic breast cancer [4]. Breast cancer is fairly heterogeneous; gene-expression profiling of breast cancer revealed six intrinsic molecular subtypes: luminal A (estrogen receptor (ER)+, progesterone receptor (PR)+, human epidermal growth factor receptor 2 (HER2)−, and Ki67−), luminal B (ER+, PR+, HER+/−, and Ki67+), HER2+, basal-like subtype (ER−, PR−, and HER2−), normal breast-like, and claudin-low (low expression of cellular adhesion genes) [5,6,7]. Triple-negative breast cancer (TNBC) belongs to either the basal-like or claudin-low subtypes [7]. Breast cancer subtypes differ in terms of clinical relevance, patterns of gene expression, selection of therapeutic strategies, responses to treatment, and prognosis [5,8,9]. Therefore, knowledge of the specific breast cancer subtype is important in guiding treatment decisions and predicting prognosis. 

Breast cancer screening and early diagnosis play critical roles in improving therapeutic outcomes, leading to a better prognosis for breast cancer patients [10]. Mammography is currently the main commercially available detection method for breast cancer; however, it has numerous well-known limitations including low sensitivity of 25~59% for detecting cancer in dense breasts, which present commonly in younger women, as well as high rates of false-negatives and false positives, and 1–10% overdiagnosis [11,12,13]. Therefore, the effective management of breast cancer during therapy or early detection depends on the availability of reliable noninvasive diagnostic, prognostic, and predictive biomarkers [14,15]. In addition, an increasing number of patients demand personalized or precise treatments; hence, the identification of novel biomarkers for diagnosis and prognosis and the development of new drugs is urgently required. 

Biomarkers for cancer include substances released from the cancer cells themselves or by other tissues in response to tumors as well as physiological markers that can be visualized using imaging technology or detected by molecular technology [16,17]. Biomarkers are objective and quantifiable evaluations of biological states or diseases that can predict tumor behavior, prognosis, or treatment responses, thus playing an important role in the management of breast cancer [18,19]. They must be validated by human samples to ensure that they reflect the clinical outcome [20,21]. Because tumor cells are highly heterogeneous, a single biomarker might not have sufficient sensitivity and specificity to accurately predict cancer progression and metastasis, and a combination of multiple markers is more appealing. 

With the rapid advancement of molecular signaling pathways and genetic signatures, including immunohistochemistry, next-generation sequencing, and targeted multigene, numerous clinically relevant biomarkers in tissue and/or blood (liquid biopsies) have been reported to aid in determining the risk of metastasis, prognosis, recurrence, treatment guidance, and drug resistance in breast cancer. Some of these have been used clinically [19,22,23,24]. However, they lack specificity and sensitivity. Therefore, the identification of novel and effective biomarkers is urgently required. In addition, there is an emerging development of immunotherapies for breast cancer, and it is important to identify reliable biomarkers for predicting who will benefit from immunotherapies.

In this review, we summarize and focus on the recent discovery of promising biomarkers for the diagnosis and prognosis of breast cancer and provide their implications in therapeutic strategies. 

## 2. Types of Biomarkers

Biomarkers used in cancer range from macromolecules, such as DNA, genetic mutations, RNA, and proteins to whole cells (Table 1 and Table 2). They can circulate in the blood as circulating mRNA, circulating free DNA, and circulating tumor cells, making liquid biopsies attractive for clinical use [17,25,26]. Two types of biomarkers are used for cancer treatment outcome: prognostic biomarkers are associated with clinical outcome and can inform whether a patient should be treated, and predictive biomarkers to guide a treatment that is effective only in a subtype of breast cancer [27,28,29]. Some biomarkers are already available in clinical practice, whereas some biomarkers have been validated in mouse models or clinical trials.

### 2.1. Proteins

Proteins involved in cell proliferation and angiogenesis are often involved in tumorigenesis when deregulated. Circulating proteins in the blood are good candidates as biomarkers for tumor detection, including breast cancer. Identification of protein biomarkers can be systematically screened by integrating blood proteomics, such as enzyme-linked immunosorbent assay (ELISA) and mass spectrometry to compare the physiological and pathological conditions [16]. The identified protein markers must be validated using human samples or clinical trials. 

Alternatively, antibodies that target proteins encoded by oncogenes and tumor suppressor genes and produced by the cells of the organism are potential biomarkers for early detection of breast cancer [113]. As such, many studies have identified tumor-associated antigens (TAAs) recognized by autoantibodies in the blood samples of patients. The integration of the newly developed technologies of genomics, proteomics, high-throughput technologies, and immunological methods has greatly progressed in this field [114].

### 2.2. Non-Coding RNAs (ncRNAs)

MicroRNAs (miRNAs or miRs) are short (20–25 nucleotides), endogenous, single-stranded, highly conserved, non-coding RNAs that downregulate post-transcriptional gene expression by controlling diverse cellular pathways, by mRNA degradation, or translation silencing [115,116]. Dysregulation of miRNAs plays prominent roles in tumorigenesis, progression, apoptosis, invasion, and metastasis as oncogenes (oncomiRNAs) and tumor suppressors [117,118].

As miRNAs are stable and detectable in tumor tissues [119], serum, plasma, and the saliva of patients, circulating miRNAs can be biomarkers for noninvasive early detection, diagnosis, and prognosis of breast cancer [120,121,122]. Apoptotic and necrotic cells release miRNAs into the bloodstream [123]. miRNAs can also be circulated in the blood in two forms: cell-free, related to Argonaute 2 (Ago2), or encapsulated in membrane vesicles, microvesicles, or exosomes [124,125]. miRNA profiling studies could systematically identify miRNAs that are dysregulated during breast cancer metastases and stratify breast cancer patients for different treatments, reinforcing the potential of miRNAs as diagnostic and prognostic biomarkers [126]. A previous study revealed the diagnostic, predictive, and prognostic values of deregulated miRNAs in breast cancer [127]. 

In contrast to linear RNAs terminated with 5′ caps and 3′ tails, circRNAs, and errors in RNA splicing are single-stranded covalently closed circular transcripts [128,129]. Emerging evidence suggests that circRNAs are involved in the pathogenesis of various diseases, including cancer [130,131]. In particular, circRNAs play critical roles in tumorigenesis, metastasis, and drug resistance [131]. 

### 2.3. DNAs

Gene mutations are often involved in tumorigenesis. Additionally, most DNA molecules involved in cancer development are through alterations in the epigenome, resulting in differential gene expression without changing the DNA sequence. DNA methylation is one of the most important epigenetic mechanisms in cancer because it influences gene transcriptional activities by epigenetic silencing of tumor suppressor genes via hypermethylation at the CpG regions and activating oncogenes by gene-wide hypomethylation [16].

### 2.4. Exosome

Exosomes are nano-sized (30–100 nm), extracellular membrane-bound vesicles that are actively secreted by cancer cells and adjacent cells in the tumor microenvironment (TME) [125,132]. They are enclosed in a lipid bilayer consisting of phosphoglycerides, ceramides, sphingolipids, and cholesterols [133] and contain a wide range of molecules, including DNA, carbohydrates, proteins, peptides, lipids, mRNAs, miRNAs, and other types of ncRNAs [134]. Similar to miRNAs, exosomes are found in several human body fluids, such as blood, saliva, urine, and breast milk [133,135]. When secreted, exosomes bind to recipient cells via receptors and transfer intra-exosomal ingredients [136]. These unique properties make exosomes attractive biomarkers for noninvasive cancer diagnosis.

Exosomes can crosstalk between tumor cells and normal or cancer-related stromal cells to promote tumor growth, angiogenesis, immunosuppression, and metastasis [100,137]. Regarding cancer metastasis, exosomes contain different integrin proteins showing different preferences for metastatic organs [138], suggesting that exosomal integrins are possible biomarkers for predicting metastasis in multiple organs. However, all these need to be validated in both preclinical and clinical settings [24]. Additionally, tumor exosomes have been demonstrated to be delivered into various distant organs before cancer cell dissemination to create a pre-metastatic niche in the lung, bone, and liver [138].

### 2.5. Cells

#### 2.5.1. Tumor-Infiltrating Lymphocytes (TILs)

Various types of immune cells present in the TME, including cytotoxic T cells, T helper (Th) cells, natural killer (NK) cells, dendritic cells, and macrophages, are collectively called tumor-infiltrating lymphocytes (TILs) [139,140]. TILs are correlated with prognosis and response to therapy in cancer [139,141]. In breast cancer, a growing number of studies have evaluated the significance of TILs as prognostic and predictive markers and their specific subsets. Although evaluation of the overall frequencies of TIL based on H&E staining upon routine diagnostics is feasible [32,107], TILs are highly heterogeneous and vary across different molecular subtypes of breast cancer, and not all types or subsets of immune cells are associated with improved outcomes. Thus, this technique cannot accurately evaluate different immune subsets. Alternatively, more advanced techniques, including polymerase chain reaction (PCR), flow cytometry, and immunogenomics, will be required to assess TILs more accurately [142,143,144,145,146].

#### 2.5.2. Tumor-Associated Stromal Cells

Tumor-associated stromal cells are cancer-associated fibroblasts and macrophages, which play important roles in breast cancer progression [147]. Accumulating evidence indicates that stromal and immune gene signatures are potential prognostic or predictive biomarkers for breast cancer. However, immune gene signatures have not been included in the current multigene assays, and currently no commercial assay is available [148]; the development of stromal or immune gene signatures that reflect the TME may contribute to better prognostic or predictive biomarkers for breast cancer [105].

## 3. Biomarkers for Cancer Risk

There are limited studies on biomarkers utilized in cancer risk. A few recent examples exist. Investigating the role of inflammation in breast cancer risk might reveal useful inflammatory biomarkers in the blood. Only one inflammatory marker, C-reactive protein (CRP), a sensitive and widely used systemic marker for inflammation, has been extensively studied and shown to be correlated with breast cancer risk [149,150]. Previous studies disclosed stronger correlations between CRP and breast cancer risk in the first few years of follow-up, which might suggest consequences rather than causes of underlying cancer [151,152]. However, a meta-analysis by Chan et al. found that positive correlations remained in studies that excluded early years of follow-up [153]. Because of inconsistencies across studies, further research should be performed to confirm the positive association between blood CRP levels and breast cancer risk [149]. There is limited evidence showing that other inflammatory biomarkers and comprehensive panels of biomarkers are correlated with breast cancer development. Thus, more prospective studies investigating systemic blood inflammatory biomarkers are required to establish a link with breast cancer risk. This will help discover potential immune biomarkers to prevent breast cancer [149].

The study by Fan et al. [96] identified peptides KNG1_K438-R457_, derived from kininogen-1 (KNG1), and C3f_S1304-R1320_, part of complement C3 (C3), as putative peptide candidates for differentiating *BRCA1* mutant breast cancer from sporadic breast cancer and cancer-free *BRCA1* mutant carriers. Thus, the expression and activity of both peptides were associated with *BRCA1* status. This approach can predict the risk of cancer. However, this needs to be validated by more prospective studies. C3 plays a critical role in the complement system contributing to innate immunity [154], which again highlights the importance of immunity in breast cancer prediction. 

Furthermore, it was disclosed that the inclusion of sex steroids and hormones, such as dehydroepiandrosterone sulfate, estradiol, testosterone, estrone, sex hormone-binding globulin (SHBG), IGF-I, IGF-binding protein 3, or prolactin, might improve the prediction of risk for invasive breast cancer for pre- and postmenopausal women [91].

Investigating the clinical benefits of cancer metabolism is required to identify metabolic pathways that limit tumor development [155]. Recent studies have revealed that certain oxysterols show tumor-promoting as well as tumor-suppressing properties [156]. Clinical studies from the Heidelberg cohort from the European Investigation into Cancer and Nutrition (EPIC) showed that higher circulating 27-hydroxycholesterol (27-HC) was associated with lower breast cancer risk in postmenopausal women, indicating that 27-HC may prevent breast cancer [97,98].

## 4. Biomarkers for Cancer Diagnosis

Early detection is critical but a difficult task for managing cancer. If tumors can be diagnosed before cancer cell metastasis, the mortality rate will be greatly reduced. However, there are currently no reliable technologies for screening tumors before clinical symptom manifestation. Considering the currently utilized cancer diagnostic tools, such as CT, MRI, and histopathology, which are invasive or expensive; mammography has relatively low resolution and sensitivity. Therefore, minimally invasive and inexpensive methods are required [129]. Therefore, considerable effort has been devoted to identifying clinically useful biomarkers for cancer diagnosis. 

### 4.1. Proteins

Currently, carbohydrate antigen 15–3 (CA15-3) is the most frequently used serum marker for routine breast cancer screening, monitoring, and follow-up of breast cancer patients [157]. However, the traditional serum tumor biomarkers for breast cancer, such as carcinoembryonic antigen (CEA), cancer antigen 125 (CA125), and CA15–3, show low sensitivity and are frequently utilized for follow-up monitoring instead of early diagnosis [158]. Hence, there is an urgent need to identify novel serum tumor biomarkers for breast cancer screening [80]. The combination of all four proteins, tumor abnormal protein (TAP) + CEA + CA125 + CA15-3, revealed the highest sensitivity for breast cancer diagnosis (21.84%), and might thus be auxiliary for early detection of breast cancer [80].

Recently, a panel of trefoil factor (TFF) 1, TFF2, and TFF3 has been reported as a potential biomarker for breast cancer screening because of its impressive ability to differentiate between breast cancer patients and healthy individuals; serum TFF1 and TFF3 levels were demonstrated to be significantly higher and the serum TFF2 level was significantly lower in breast cancer patients than in healthy individuals [81]. Trefoil factors are secretory proteins expressed in the gastrointestinal mucosa [159]. TFF1 was recently reported to inhibit cell growth, migration, and invasion of breast cells in vitro and has also been suggested to be a prognostic biomarker for breast cancer [160]. In a study by Giussani et al. [86], plasma samples from healthy controls and patients with malignant or benign breast disease were tested for the expression of extracellular matrix (ECM) molecules, collagen 11a1 (COL11A1), collagen oligomeric matrix protein (COMP), and collagen 10a1 (COL10A1). Notably, the combination of COL11A1, COMP, and COL10A1 was identified as a promising biomarker to differentiate breast cancer patients from the benign controls. Therefore, circulating COL11A1, COMP, and COL10A1 could be used as biomarkers to diagnose breast cancer. This study addressed the importance of ECM molecules in breast cancer development. Moreover, pleiotrophin (PTN), a circulating protein biomarker with adequate differentiating ability, is superior to CEA and CA15-3 [82]. PTN is a growth factor that regulates several cellular functions, and its high expression is associated with many cancer types [82]. Thus, PTN may be a potential biomarker for breast cancer diagnosis. In two-marker models, such as the combination of plasma human epididymis secretory protein 4 (HE4) and miR-127-3p, the expression of plasma miR-127-3p in breast cancer patients was significantly higher than that in benign breast tumors and healthy controls (both *p* < 0.001) [83]. It has been reported that HE4 plays a critical role in the diagnosis of several tumor types, including breast, lung, and ovarian cancers [83]. Others are the combination of vascular endothelial growth factor (VEGF) with CA 15–3 [84] and the combined human anterior gradient (AGR) 2 and AGR3 biomarker panel [85]. In addition, CA15-3 was also included in the diagnostic panel consisting of four protein peaks [m/z 3972, 6850, 8115 (Bc2), and 8949 (Bc3)] utilized to differentiate 62 breast cancer patients with invasive ductal carcinoma from 16 healthy controls and 31 patients with benign breast diseases [87]. Notably, the resultant four peak panel, together with CA15-3, was proven to show high sensitivity and specificity for breast cancer diagnosis. However, further large-scale studies are needed to verify these results.

To further identify the subtype of breast cancer, such as TNBC, serum apolipoprotein C-I (apoC-I) was identified and proven to be upregulated in TNBC compared with both non-TNBC and the controls, including benign breast disease and healthy subjects. Hence, it has shown potential as a diagnostic and prognostic biomarker for TNBC [161]. In contrast, Sun et al. [162] discovered that APOC1 reduced significantly in breast cancer, and its expression was downregulated in the TNBC and pre-surgery groups compared to that in the non-TNBC and post-surgery groups. Therefore, there are discrepancies between these two studies; further investigations involving more samples are necessary to confirm the role of apoC-I in TNBC.

Apart from the single-marker signature, a common strategy adopted in biomarker research to improve diagnostic accuracy is the incorporation of novel autoantibodies with classical tumor markers [16]. Autoantibodies or tumor-associated autoantibodies can be potential biomarkers for early breast cancer diagnosis based on the concentration, which may precede the clinical confirmation of cancer by months to years, as the detection of autoantibodies can be performed earlier than the originating TAA assays [163]. For example, using serological analysis of recombinant cDNA expression libraries (SEREX) in combination with phage display technology, a panel of serum autoantigens consisting of galectin 3 (LGALS3), prohibitin 2 (PHB2), MUC1, glycerol kinase 2 (GK2), and CA 15–3 demonstrated better diagnostic values for early-stage breast cancer compared to anti-CA 15–3 alone [88]. However, this panel of complex autoantigens needs to be validated using more breast cancer samples. SEREX was also used to identify a combination of six antigens: RAD50 double-strand break repair protein (RAD50), par-3 family cell polarity regulator (PARD3), secreted phosphoprotein 1 (SPP1), SAP30 binding protein (SAP30BP), kinesin family member 15 (NY-BR-62), and NY-CO-58, which could differentiate breast cancer patients from healthy subjects [89]. Investigating autoantibodies could provide valuable information for identifying novel therapeutic targets and diagnostic biomarkers for breast cancer. However, it should be noted that in a recent meta-analysis by Xia et al. [164], there are considerable inconsistencies in terms of sensitivity and specificity of these autoantibodies as potential diagnostic biomarkers for breast cancer. These differences may be due to different assays and platforms, different experimental procedures, and different patient populations. Therefore, it should be confirmed through more studies using more samples before we can accept the diagnostic or predictive values of these autoantibodies [165]. 

Henderson et al. [90] evaluated for the first time the independent and combinatorial contribution of serum protein biomarkers and tumor-associated autoantibody expression data for the identification of breast cancer. Importantly, when combining serum protein biomarkers and tumor-associated autoantibodies, the clinical sensitivity and specificity for detecting breast cancer improved to 81.0% and 78.8%, respectively. These data revealed the advantage of combining serum protein biomarkers and tumor-associated autoantibodies data.

### 4.2. miRNAs

The miRNAs reported to be significantly upregulated in the plasma, serum, and tissues of breast cancer patients include miR-141, miR-200a/b/c, miR-203, miR-210, miR-375, miR-801 [46,166], miR-10, miR-155, miR-191, miR-382 [167], miR-451 [168], miR-21 [46,169], miR-199a-5p [170], miR-195 [171], miR-148b, miR-376c, miR-409-3p, miR-801 [172], and miR-1204 [54], whereas miR-181a [173], miR768-3p [166], miR-145 [46,168], miR-139-5p [46], miR-92a [169], miR-99a, miR-195, miR-497, and miR-205 [46] were found to be downregulated in breast cancer patients when compared to their levels in healthy controls. Some of these miRNAs have revealed sufficient sensitivity for early detection and/or prognosis for breast cancer in preliminary results, but require further validation through larger-scale investigation [24].

MiR-21 is the most consistently reported miRNA to be upregulated in breast cancer [46] and plays important roles in cancer diagnosis and prognosis as well as in cancer development. Recently, miR-21 and miR-221 differentiated breast cancer cases from patients with benign tumors and healthy controls [47]. Six miRNA signatures, miR-21, miR-221, miR-210, miR-195, miR-145, and Let-7a, may serve as minimally invasive biomarkers for the early detection of TNBC [48]. Furthermore, the expression of plasma exosomal miR-21 and miR-1246 was significantly higher in breast cancer patients than in healthy control subjects. Thus, the combination of exosomal miR-21 and miR-1246 generated a moderate diagnostic model [50]. In addition, a comprehensive Asian study identified a five-miRNA signature, miR-1246, miR-1307-3p, miR-4634, miR-6861-5p, and miR-6876-5p from 4630 serum samples, which could detect early-stage breast cancer with a high sensitivity of 97.3% [51]. Nevertheless, a prominent limitation of this study is that the results were based primarily on microarray analysis, and only miR-1246 was validated by quantitative real-time PCR [51]. Consistently, a recent meta-analysis combining six previous publications on miR-21 from both Caucasian and Asian cohorts has further confirmed miR-21 as a potential biomarker for the early diagnosis of breast cancer [174].

Besides miR-21, an enormous number of miRNAs have been identified as single markers or in combination for the diagnosis of breast cancer. Xu et al. discovered that miR-154 was downregulated in breast cancer tissues and its role in targeting E2F transcription factor 5 protein (E2F5) [175]. In a recent study, high miR-1204 expression was correlated with TNM stage, differentiation grade, and lymph node metastasis. Furthermore, breast cancer patients with higher tissue and serum miR-1204 expression had shorter overall and disease-free survival times. Hence, miR-1204 could be a novel noninvasive prognostic/diagnostic marker for the early detection of breast cancer [54]. The eight-marker signature consisting of miR-16, let7d, miR-103, miR-107, miR-148a, let-7i, miR-19b, and miR-22 has been reported for the early detection of breast cancer, including breast cancer detection in younger women [52], and also for being a better screening method than mammography and CA 15–3 assays [52]. miR-140 and miR-196a showed decreased and increased expression in breast cancer samples compared to adjacent non-tumor tissues, respectively (*p* < 0.001). These results indicate that both miR-140 and miR-196a are potential biomarkers for the diagnosis and management of breast cancer. Nonetheless, the limitations of the present study were that they could not investigate the correlation between miRNA expression and the survival rate because it requires a 5-year follow-up of breast cancer patients [56]. Even though a combination of multiple biomarkers has a better diagnostic value than a single marker [176], a minimal number of markers, which can show a high diagnostic value, should be selected, as it is more cost-effective for population-wide screening [16]. 

In inflammatory breast cancer tissues, a rare type of breast cancer, the expression of miR-181b-5p was significantly upregulated, whereas miR-200b-3p, miR-200c-3p, and miR-203a-3p were significantly downregulated. These identified miRNA signatures can be utilized individually to differentiate inflammatory breast cancer from non-inflammatory breast cancer patients. It is important to note that the combination of miR-181b-5p, miR-200b-3p, and miR-200c-3p robustly improved the accuracy of this differentiation. These results indicate that the combination of miR-181b-5p, miR-200b-3p, miR-200c-3p, and miR-1-3p may be promising diagnostic biomarkers for patients with inflammatory breast cancer [55]. However, their findings should be verified comprehensively in a prospective large cohort of patients with inflammatory breast cancer, and their roles as therapeutic targets should be further examined. 

Despite the numerous studies identifying potential miRNA biomarkers for breast cancer, there is a large discrepancy in the miRNA signature identified in these studies. The possible reasons might be a lack of standardization in sample preparation and storage and the different methods utilized for identifying the targeted miRNA as well as different methods for miRNA validation and quantification.

### 4.3. circRNAs

As circRNAs have received more attention for their involvement in tumor pathogenesis, the diagnostic value of circRNAs has gained more attention recently. Accumulating evidence has demonstrated that higher circRNA levels are detected in normal breast mammary tissues than in tumor tissues. Lu et al. [74] screened the circRNA expression profiles in breast cancer and adjacent normal tissues using circRNA microarray analysis and showed that hsa_circ_103110, hsa_circ_104689 and hsa_circ_104821 levels were upregulated in breast cancer tissues, whereas hsa_circ_006054, hsa_circ_100219, and hsa_circ_406697 were downregulated. Thus, combining hsa_circ_006054, hsa_circ_100219, and hsa_circ_406697 showed good diagnostic values. Similarly, Yin et al. discovered that 19 circRNAs were upregulated and 22 were downregulated in the plasma of breast cancer patients compared to the healthy controls [73]. Further analysis revealed that hsa_circ_0001785 in the plasma showed higher diagnostic accuracy than CEA and CA15-3. Moreover, hsa_circ_0001785 plasma levels were closely associated with histological grade (*p* = 0.013), TNM stage (*p* = 0.008), and distant metastasis (*p* = 0.016), suggesting a potential biomarker for the diagnosis of breast cancer [73]. 

### 4.4. DNAs

Changes in DNA methylation are one of the most common molecular alterations in carcinogenesis. The methylated promoters of tumor suppressor genes, adenomatous polyposis coli (*APC*), and retinoic acid receptors-β2 (*RARβ*_2_) were observed in 93.4% and 95.6% of serum samples, respectively, from breast cancer patients, but were not detected in healthy subjects. Both methylated genes outperformed the traditional markers, CEA and CA 15–3, for detecting early-stage breast cancer, low-grade tumors, and TNBC [36]. Using the human methylation DNA analysis BeadChip, Yang et al. identified that peripheral blood S100 calcium-binding protein P (*S100P*) and hyaluronoglucosaminidase 2 (*HYAL2*) hypomethylation was associated with breast cancer. Both genes with decreased methylation were demonstrated to be promising blood-based biomarkers for detecting breast cancer, especially in the early stages, including in younger women [37,38]. S100 proteins regulate numerous cellular processes, such as cell cycle progression and differentiation, and enhance cell migration [177]. *HYAL2* encodes a lysosomal hyaluronidase, which degrades hyaluronan, one of the major glycosaminoglycans in the extracellular matrix. Hyaluronan is suggested to be involved in cell proliferation, migration, and differentiation [38]. Therefore, these results indicate the importance of *S100P* and *HYAL2* in breast cancer development. A meta-analysis proved that promoter methylation of *14-3-3 σ*, *a* tumor suppressor, may be a useful blood-based diagnostic biomarker for breast cancer diagnosis [35]. 

Recently, the association between methylation of the long noncoding RNA 299 gene (*LINC00299*) and TNBC risk was evaluated using a prospective study design. There was no correlation between the *LINC00299* methylation levels and TNBC overall (*p* = 0.062). However, subgroup analysis revealed higher methylation levels in the young TNBC patients compared to the controls [age 26–52 (*p* = 0.0025) and age 22–46 (*p* = 0.001), respectively]. Therefore, the results suggest that the *LINC00299* methylation level may be a potential biomarker for the early detection of TNBC in young women. The different results obtained in the age subgroups might be due to differences in the molecular features of TNBC between younger and older women [39]. This needs to be further verified through larger studies.

A highly sensitive circulating cell-free DNA (cfDNA) system for the early diagnosis of breast cancer by incorporating epigenetic biomarkers and droplet digital methylation-specific PCR (ddMSP) has been developed [178]. The best detection model adopted four methylation markers consisting of Ras-specific guanine nucleotide-releasing factor 1 (*RASGRF1*), carboxypeptidase X (*CPXM1*), Homeobox protein Hox-A10 (*HOXA10*), and Dachshund homolog 1 (*DACH1*). This novel epigenetic-marker-based system could discriminate breast cancer patients from healthy controls with high accuracy, indicating the potential use of this system for breast cancer screening [178]. 

### 4.5. Exosomes

Developmental endothelial locus-1 protein (Del-1) [100] and fibronectin [99], the surface proteins on circulating extracellular vesicles (EVs), are promising candidates for cancer detection. Fibronectin levels increased significantly (*p* < 0.0001) at all stages of breast cancer and returned to normal levels after tumor removal. The diagnostic accuracy for fibronectin detection in extracellular vesicles was also better than that for fibronectin detection in plasma [99]. Fibronectin is an extracellular matrix protein that binds multiple integrins and activates various signaling proteins, such as focal adhesion kinase (FAK), Src, and Akt [99]. This again emphasizes that extracellular matrix proteins are associated with breast cancer. The elevated level of Del-1 in circulating exosomes of breast cancer patients (*p* < 0.0001) yielded an outstanding diagnostic performance in differentiating early-stage breast cancer patients from the controls [100].

## 5. Biomarkers Involved in Cancer Progression/Proliferation

Cancer is a genetic disease, and its development and proliferation depend on the regulation of many proteins and miRNAs [179]. Human serum contains circulating proteins, miRNAs, and circRNAs, which could be potential therapeutic targets for breast cancer.

### 5.1. miRNA

As mentioned earlier, miR-21 is overexpressed in breast cancer (Section 4.2, [46]). miR-21 promotes the transformation and development of breast cancer by inhibiting the expression of programmed cell death protein 4 [49]. MiR-182, a member of the miR-183 family, is packed into exosomes. It has been shown that the high levels of exosomal miR-182 in breast cancer may contribute to tumor progression [59]. In contrast, many miRNAs, such as lethal-7 family (let-7), miR-26b, miR-124, miR-125a/125b, miR-205, and miR-206, were demonstrated as tumor-suppressor miRNAs [58]. Ma et al. discovered that miR-26a/26b could suppress breast cancer progression by downregulating the expression of ST8 alpha-N-acetyl-neuraminide alpha-2,8-sialyltransferase 4. As a result, 26a/26b may be useful markers for breast cancer progression [57]. miR-26b was further investigated and found to inhibit TNBC cell growth by repressing the DEP domain containing 1 and downregulating FOXM1 expression [58]. Moreover, Liu et al. reported that miR-224 downregulated Wnt/β-catenin signaling by targeting frizzled 5, thereby decreasing the percentage of breast cancer stem cells and suppressing cell proliferation and migration [60]. More recently, miR-124-3p was reported to be downregulated in breast cancer cells, where it inhibited cell proliferation and metastasis by targeting N-acetylglucosaminyltransferase V (MGAT5), a tumorigenesis and metastasis-associated enzyme in breast cancer [61]. All the results mentioned above need to be further validated through larger-scale studies. 

### 5.2. circRNA

circRNAs play key roles in the regulation of the development and progression of human cancers. hsa_circ_0001982 [75] and circ-ABCB10 [77] knockdown inhibited proliferation and invasion and upregulated apoptosis of breast cancer cells by targeting miR-143 and miR-1271, respectively. In TNBC, circGFRA1 is overexpressed in breast cancer and is positively associated with poorer survival by binding to miR-34a [78]. Although these results are promising, further studies are required to validate the circRNAs identified before clinical use. Additionally, enhanced circRNA-000911 expression inhibited cell proliferation and migration and induced apoptosis of breast cancer cells. miR449a antagonized circRNA-000911 to regulate breast cancer cell progression via the Notch1 and NF-κB signaling pathways. Thus, circRNA-000911 may be a promising therapeutic target for breast cancer, and overexpression of circRNA-000911 may provide a future direction for the development of novel therapeutic strategies for breast cancer [76]. 

## 6. Biomarkers Involved in Cancer Recurrence and for Cancer Prognosis

Evaluation of cancer prognosis also plays an important role in the early stage of treatment and decision making in the following intervention. Because breast cancer is extremely heterogeneous, a single biomarker might not be suitable for the accurate evaluation of cancer prognosis, and multiple gene signatures or combinations of biomarkers are more clinically applicable for cancer prognosis. A number of commercial products incorporate multiple genes and/or proteins that are already available for clinical use, such as Oncotype DX [180], MammaPrint [181], Breast Cancer Index [182], Rotterdam Signature [183], Prosigna [23], EndoPredict [184], and GenesWell BCT [185]. Most of these have been validated or validated by clinical trials. Here, we present recent discoveries of biomarkers identified for cancer prognosis, some of which have not been validated by clinical trials. 

### 6.1. DNAs

Mutations in the ER gene (*ESR1*) were identified in 39.1% and 25.3% of patients from the Study of Faslodex Versus Exemestane with or without Arimidex (SoFEA) trial and the PALOMA-3 trial [186], respectively. In the SoFEA cohort, patients with *ESR1* mutations had better progression-free survival (PFS) when treated with fulvestrant than those treated with exemestane (*p* = 0.02). In contrast, the PFS of patients with wild type *ESR1* was similar in both treatments (*p* = 0.77). In the PALOMA-3 cohort, both PFS of *ESR1* mutant (*p* = 0.002) and *ESR1* wild type (*p* < 0.001) were improved by treatment with fulvestrant plus palbociclib compared with fulvestrant plus placebo. These results suggest that *ESR1* mutation analysis in plasma with progression on prior endocrine therapy may help determine the choice of further endocrine therapy [40]. However, this needs to be confirmed through further prospective studies. In the BOLERO-2 double-blind phase III study, the combination of exemestane and everolimus was compared to exemestane alone in ER-positive aromatase inhibitor-treated metastatic breast cancer. The two most frequent *ESR1* mutations, Y537S and D538G, were identified in cfDNA from plasma specimens (28.8%). These *ESR1* mutations were correlated with shorter overall survival, indicating a more aggressive phenotype, thereby predicting the prognosis of metastatic breast cancer [41]. However, it is important to consider the ease and feasibility with which this biomarker could be obtained. The *TP53* mutation also showed better prognosis in metaplastic breast cancer, as it was correlated with better recurrence-free survival (RFS, *p* = 0.03) and OS (*p* = 0.06) compared to mutations in the PI3K/Akt/mTOR pathway [43]. However, this needs to be further validated in a large-scale study.

In a recent study, Wang et al. [44] developed a 14-gene prognostic signature containing *PFKL*, *P4HA2*, *GRHPR*, *SDC3*, *PPP1R15A*, *SIAH2*, *NDRG1*, *BTG1*, *TPD52*, *MAFF*, *ISG20*, *LALBA*, *ERRFI1*, and *VHL*. The hypoxia-related signature successfully predicted the overall survival rates of breast cancer patients (*p* < 0.001, *p* = 0.007, *p* = 0.021, and *p* < 0.001) for the TCGA dataset and Gene Expression Omnibus databases, GSE10886, GSE20685, and GSE96058, respectively. Therefore, the 14-gene hypoxia-related signature could be a promising biomarker for prognosis and targets for breast cancer. However, this result should be further confirmed using cell lines, animal experiments, and human samples. In recent years, multigene tests have been developed for the early diagnosis and prognosis prediction of breast cancer. Liu et al. [45] developed a 28–5′-C-phosphate-G-3′ (CpG) DNA methylation panel for the diagnosis and prognosis of breast cancer patients. Higher methylation risk was correlated with poor overall survival in breast cancer patients and tumor heterogeneity. Furthermore, DNA methylation panels could differentiate breast cancer patients into different survival groups. However, the survival differences between the low-risk and high-risk cohorts of the different subgroups were not statistically significant. Importantly, the multi CpG methylation panel was demonstrated to have excellent performance compared with other well-known multi-biomarkers [45]. However, the results still need to be validated through large-scale prospective studies before the findings can be applied clinically.

### 6.2. miRNA

As “precision medicine” is gaining more attention, circulating miRNAs in the blood have been utilized as diagnostic and prognostic biomarkers. A systematic review and meta-analysis showed that downregulation of miR200c and miR489 was correlated with better prognosis; similarly, both upregulated miR484 and miR4443 were associated with better prognosis. In contrast, upregulated miR520h and miR125b were correlated with poor prognosis [64]. Moreover, circulating miR21 and miR125B were correlated with neoadjuvant chemotherapy response and disease-free survival (DFS) and thus are regarded as novel noninvasive prognostic biomarkers for breast cancer [65]. Another study revealed that circulating miR-106b is a putative prognostic biomarker because its expression is associated with tumor size, Ki67 expression, lymph node metastasis, and shorter OS and DFS [66]. Interestingly, precursor miRNA, pre-miR-488, may be a novel prognostic biomarker that predicts disease recurrence in breast cancer [67].

### 6.3. Immune Gene Signatures and Immune Cells

Analysis of both tumor cells and the surrounding stromal cells may identify possible prognostic biomarkers, as both tumor cells and the surrounding stromal cells play a role in prognosis. In general, upregulation of immune genes was correlated with favorable prognosis in HR− breast cancer patients [105]. A recent study also discovered that increased expression of immune response-related genes, *BTN3A2*, *CD2*, and *TRBC1* was correlated with favorable prognosis of HR−/HER2+ breast cancer, indicating the importance of immune response-related genes in predicting the treatment outcome for this subtype [30]. Furthermore, 17 immunity genes, *APOBEC3G*, *CCL5*, *CCR2*, *CD2*, *CD27*, *CD3D*, *CD52*, *CORO1A*, *CXCL9*, *GZMA*, *GZMK*, *HLA-DMA*, *IL2RG*, *LCK*, *PRKCB*, *PTPRC*, and *SH2D1A*, were identified from the Affymetrix gene expression dataset for significant prognostic stratification of ER- breast cancer and highly proliferative breast cancers. Patients with increased immunity genes had significantly favorable outcomes [31]. In particular, *CD2* was incorporated into these two studies, demonstrating its importance in prognosis. 

Apart from immune response-related genes, adaptive immune cells, including T and B cells, are also associated with favorable prognosis in lymph node–breast cancer patients with stages I–III [101]. Furthermore, a number of studies have demonstrated that high frequencies of CD8 effector T cells and T helper type-1 gene signatures (Th1: *IFNG*, *STAT1*, *GRZM*, *CXCL9*) are associated with better outcomes, particularly in ER-tumors [32,33,34]. Similarly, high numbers of regulatory T cells (Tregs) in tumor tissue and blood are favorable prognostic factors in the HER2+/ER– subtype, whereas Tregs are correlated with poor outcome in ER+ breast cancer [34]. 

TILs are gaining attention for their prognostic value in breast cancer. A high frequency of TILs has been observed in the more aggressive breast cancer subtypes, including the ER subtypes, that is, HER2 and basal, highly inflamed tumors, and the highly proliferating luminal B subtype, but are low in the less aggressive luminal A subtype. Therefore, the TIL frequency was found to be a promising prognostic marker and associated with a favorable prognosis [33,106,108,187,188,189]. High numbers of TILs and CD8 T cells were shown to be predictive of the response to checkpoint inhibitors, pembrolizumab, following irradiation and chemotherapy for metastasized TNBC [104]. High TILs have also been demonstrated to be associated with improved responses to trastuzumab or lapatinib in HER2+ breast cancer [188,190,191]. However, a recent study revealed that high TIL levels were associated with shorter overall survival in HR+/HER2− breast cancer, while high TIL levels were correlated with better outcomes in TNBC [192]. The differences in the prognostic values of TILs in different breast cancer types may be due to different immune cell compositions. Further investigation of the interactions between immune cells and tumor cells in different breast cancer cell types is required. 

### 6.4. Proteins

Recently, Kim et al. [95] discovered a novel TNBC-specific RNA-binding protein (RBP), NONO, regulating signal transducer and activator transcription 3 (STAT3) to exert its oncogenic function. Both NONO and STAT3 levels were inversely correlated with the response to chemotherapeutic drugs and the prognosis of TNBC. Notably, auranofin, a potential NONO inhibitor, identified via high-throughput drug screening, inhibits cell growth in TNBC. Therefore, these findings indicate that RBP may be a novel therapeutic target for treating TNBC.

## 7. Biomarkers Involved in Cancer Metastasis

Breast cancer is a metastatic disease that invades various organs such as the lung, bone, liver, and brain. Metastasis to different organs often results in different clinical outcomes. Therefore, biomarkers for organotropism have been intensively studied to predict organ preference [24]. Genes that mediate breast cancer metastasis to the bone [193], lung [194], and brain [195] have been identified. Some affect the overall metastasis, whereas others play organ-specific roles in metastasis, which regulate the interaction between cancer cells and the microenvironment of target organs [24]. Importantly, identifying and inhibiting biomarkers involved in the metastasis of cancer cells are critical for suppressing metastasis and improving clinical outcomes. The following are recent examples.

### 7.1. Chemokines

Chemokine receptor 9 (CCR9) binds to ligand chemokine 25 (CCL25) and regulates the trafficking of lymphocytes, as well as cancer cell lines. CCL25/CCR9 signaling can promote the migration and invasion of breast cancer cells to the bone by regulating numerous markers of epithelial–mesenchymal transition (EMT) [92]. However, more comprehensive investigations into this signal are required to confirm its role in bone metastasis, and it is still uncertain whether the CCR9 antibody is useful for treating breast cancer with bone metastasis.

CCL18, which is derived from tumor-associated macrophages (TAMs), via its receptor, PITPNM3, recruits inflammatory monocytes and Tregs to the breast cancer TME and induces the invasion and metastasis of breast cancer through the phosphoinositide-3-kinase (PI3K)/anti-apoptotic kinase (Akt)/GSK3β/Snail pathway [93,94]. The anti-CCL18 antibody could disrupt the GM-CSF-CCL18 feedback loop to treat cancer metastasis, especially breast cancer metastasis. Although the CCL18-PITPNM3 signaling pathway has not been revealed to play a role in bone metastasis of breast cancer, it remains unknown whether the anti-CCL18 antibody could be utilized for the treatment of breast cancer with bone metastasis [196]. Further investigation is required to confirm that CCL18 is a promising target. 

Besides these CCL25-CCR9 and CCL18-PITPNM3 chemokine signaling pathways, other chemokines, such as CCL3 and CCL22, can recruit TAMs, Tregs, and myeloid-derived suppressor cells (MDSCs) during the process of breast cancer metastasis [93]; all of these chemokine signaling pathways are promising targets for inhibiting metastatic breast cancer and are worthwhile to be further examined [196].

### 7.2. miRNAs

By downregulating E-cadherin and thus affecting EMT and breast cancer cell metastasis, miR-9 might be a valuable therapeutic target [69]. miR-205 downregulation by transglutaminase 2 (TG2) enhances breast cancer bone metastasis and invasion [70]. This was further studied using TNBC tissues. miRNA-205 could also partially suppress the migration and invasion of TNBC cells and EMT by suppressing the HMGB1-RAGE signaling pathway [71]. Furthermore, miR-628 suppressed the migration and invasion of breast cancer cells by targeting SOS Ras/Rac guanine nucleotide exchange factor 1 (SOS1), which plays an essential role in EMT. These results indicate that enhancement of these tumor suppressor miRNAs may be an effective treatment strategy against breast cancer metastasis [72].

### 7.3. Cells

Metastasis involves cancer cells and the TME; thus, non-cancer components might be novel therapeutic targets. Neutrophils [111] in the lung microenvironment have been demonstrated to support breast cancer metastasis to the lung, indicating that targeting non-cancer cell components may be a novel therapeutic approach. The interaction between cancer cells and brain-resident cells and astrocytes also facilitates metastatic colonization in the brain. The expression of phosphatase and tensin homolog (PTEN), a tumor suppressor, was silenced in cancer cells after dissemination to the brain, but not in other organs. This is epigenetically regulated by exosomal miRNAs in astrocytes. As a result, these astrocyte-derived molecules could be used as biomarkers to predict the risk of brain metastasis of breast cancer and provide new and effective anti-metastatic therapies [112].

## 8. Biomarkers Involved in Cancer Drug Resistance

Drug resistance is a major obstacle for effective breast cancer treatment; thus, identifying possible markers might solve this problem.

### 8.1. Estrogen Receptor Alpha (ESR1) Mutation

Testing blood samples from the PALOMA-3 (palbociclib combined with fulvestrant in hormone receptor-positive HER2-negative metastatic breast cancer after endocrine failure) cohort, *ESR1* mutations, especially the Y537S mutation (*p* = 0.0037), were identified in both placebo + fulvestrant and palbociclib + fulvestrant arms, suggesting that acquired mutations from fulvestrant are the major mechanisms of resistance to fulvestrant and palbociclib combination therapy [42].

Apart from ERα66 expressed in approximately 70% of breast cancers, ERα36, a variant of ERα, is expressed in ER+ and ER– breast cancer cells [197]. The roles of ERα36 in breast cancer development and drug resistance were recently reviewed by Pagano et al. [198]. Interestingly, tamoxifen, an anti-estrogen, could act as an agonist of ERα36 by activating the proliferation, invasion, and metastasis of breast cancer cells [198], which explains the drug resistance to anti-estrogens blocking the signaling pathways mediated by ERα66 in many breast cancer patients. Recently, a serum autoantibody against ERα was discovered in a large percentage of breast cancer patients and was shown to activate ERα36, contributing to tamoxifen resistance [199,200].

### 8.2. miRNA

A number of miRNAs were downregulated in breast cancer drug resistance. The expression of miR-17 and miR-20b was significantly suppressed in taxol-resistant breast cancer tissues and cells by upregulating nuclear receptor co-activator 3 (NCOA3). These results indicate that miR-17, miR-20b, and NCOA3 may be potential predictive biomarkers and therapeutic targets for taxol-resistant breast cancer [62]. Another study revealed that miR-18a was overexpressed in TNBC patients who received neoadjuvant paclitaxel, which inhibits Dicer expression and increases paclitaxel resistance in TNBC cells, and is involved in paclitaxel resistance in TNBC cells [63]. A systematic review and meta-analysis demonstrated that four miRNAs, miR-90b, 130a, 200b, and 452, were involved in chemoresistance by regulating drug-regulated cellular pathways [64]. When treated with fulvestrant, doxorubicin, or trastuzumab, miRNAs 221 and 222 were also downregulated with increased ABC transporters. Similarly, the downregulation of miRNA 320a was associated with paclitaxel treatment with downregulation of TRPC5, NFATC3, and FTS-1 genes, leading to chemoresistance. miRNAs let-7, 181a, and 145 are also majorly downregulated by treatment with doxorubicin, tamoxifen, or epirubicin, leading to chemosensitivity. In contrast, miRNA 125b was increased by treatment with tamoxifen, letrozole, anastrozole, or fulvestrant by interacting with the Akt/mammalian target of rapamycin (mTOR) pathway, ultimately causing chemoresistance [64]. In addition, multidrug resistance (MDR) is a major problem in treatment failure. miR-130b was upregulated in tumor tissues and adriamycin-resistant breast cancer cells by targeting PTEN. In particular, miR-130b contributes to MDR through the PI3K/Akt signaling pathway [68]. Therefore, miRNAs play significant roles in drug resistance and may be potential drug targets. 

### 8.3. circRNA

Using circRNA microarray expression profiles, Gao et al. [79] detected a higher expression level of hsa_circ_00006528 in adriamycin-resistant cell lines and tissues compared with the adriamycin-sensitive groups. Furthermore, the regulatory role of the hsa_circ_00006528-miR-7–5p-Raf1 axis in adriamycin-resistant breast cancer was revealed, indicating that hsa_circ_00006528 might be a possible candidate for overcoming drug resistance. Hence, circRNAs provide a novel and promising therapeutic strategy for breast cancer.

## 9. Therapeutic Implications

The currently available treatments for breast cancer are systemic, such as endocrine therapy, chemotherapy, targeted therapy, and immunotherapy, and locoregional, such as surgery and radiation therapy [201]. The heterogeneity, complexity, and metastasis of breast cancer make treatment difficult and the development of drug resistance, emphasizing the importance of discovering novel biomarkers to develop more effective personalized therapies [64,202]. The biomarkers involved in the diagnosis and prognosis of breast cancer described above are potential therapeutic targets. The therapeutic implications of biomarkers can be applied in two ways: (1) biomarkers that guide treatment decisions and (2) biomarkers that can be the targets of treatment (Table 3).

### 9.1. Endocrine Therapy

#### 9.1.1. ER

ER is the first target identified for endocrine therapy [260]. Tamoxifen is a selective estrogen receptor modulator (SERM) that acts as a competitive inhibitor of estrogen binding to estrogen receptors. Depending on the target tissue, they have mixed agonist and antagonist activities [205]. In contrast, selective estrogen receptor down-regulators (SERDs) are competitive antagonists of estrogen by binding to the estrogen receptor. Fulvestrant is approved in metastatic ER+ tumors, which downregulates ER. In contrast to SERMs, fulvestrant is a true ER antagonist with no agonistic activity [206]. Aromatase inhibitors represent another class of medicines that block the enzyme that catalyzes a key aromatization step in the synthesis of estrogen [261]. Aromatase inhibitors and tamoxifen were demonstrated to reduce recurrence rates in ER+ early breast cancer for 10 and 5 years, respectively, and reduce breast cancer mortality [207]. Aromatase inhibitors are recommended for postmenopausal women in adjuvant and metastatic settings [208].

#### 9.1.2. HER2

Trastuzumab is a humanized monoclonal antibody against the extracellular domain of HER2, resulting in the inhibition of the pathway that leads to cell growth and differentiation [209]. This is the standard of care for patients with HER2+ tumors. Other anti-HER2 therapies are currently used, such as pertuzumab (HER dimerization inhibitor), ado-trastuzumab emtansine (antibody-drug conjugates targeting HER2) [210], and lapatinib and neratinib (small molecule inhibitors against HER2) [211].

#### 9.1.3. Ki-67

The high expression of the cell cycle antigen Ki-67 is a widely utilized prognostic biomarker for chemotherapy by IHC assessment [262]. However, Ki-67 staining lacks analytical validity; thus, its performance as a prognostic biomarker remains weak, with no solid evidence for predicting adjuvant chemotherapy efficacy [263]. Only highly proliferative ER+ breast cancer with high levels of Ki-67 benefit from treatment with adjuvant docetaxel chemotherapy [203]. The use of Ki-67 to guide decisions on adjuvant chemotherapy in specific situations is endorsed by the European Group on Tumor Markers [204], yet is not supported by the American Society of Clinical Oncology or National Comprehensive Cancer Center Guidelines [264,265]. Therefore, no consensus exists yet on the effectiveness of utilizing Ki-67 to guide chemotherapy. In addition, the soluble form of transferrin receptor (sTfR), a possible indicator of bone marrow failure when receiving chemotherapy, was confirmed to be suppressed during chemotherapy, indicating that sTfR may be a potential indicator of required transfusion [266]. However, further large-scale studies in preclinical and clinical trials are required to confirm this finding.

Apart from chemotherapy, Ki-67 is an emerging biomarker for neoadjuvant endocrine therapy. In the phase III ALTERNATE study (NCT01953588), patients with ER+/Her2- invasive breast cancer received either anastrozole, fulvestrant, or a combination of both. The results showed that elevated levels of Ki-67 are associated with poor recurrence-free survival [212]. In the POETIC (Program for Enhanced Training in Cancer) trial, patients with high Ki-67 had a much higher risk of recurrence at 5 years (19.6%) in comparison with those with low Ki-67 (4.5%) [213]. Similarly, the phase II ADAPT (Adjuvant Dynamic Marker-Adjusted Personalized Therapy Trial Optimizing Risk Assessment and Therapy Response Prediction in Early Breast Cancer) trial revealed that early decrease in Ki-67 was correlated with increased pathologic complete response (pCR) to neoadjuvant treatment with either antibody-cytotoxic, anti-HER2 compound trastuzumab emtansine (T-DM1), or trastuzumab [214].

#### 9.1.4. Immune Cells

In addition, immune cells may predict clinical responses to endocrine therapy. The overall survival of HR+ post-menopausal women treated with aromatase inhibitors was associated with high numbers of TILs, especially Tregs. Thus, these markers could be utilized to guide adjuvant treatment in this breast cancer population [215]. Furthermore, there was a correlation between increased gene expression of the T-cell marker, PD-1, and response to trastuzumab treatment in HER2+ tumors [216,217]. 

### 9.2. Targeted Therapies

Most biomarkers are available for targeted therapies compared to other therapeutic strategies.

#### 9.2.1. DNAs

As mentioned above, the *ESR1* mutation is involved in drug resistance and the prognosis of breast cancer, and targeting the *ESR1* mutation might be a promising therapeutic strategy. Targeting *ESR1* mutant tumors is under investigation in metastatic breast cancer patients harboring *ESR1* mutations treated with lasofoxifene, a nonsteroidal SERM [19].

*TP53* mutations are the most common abnormalities in cancer, followed by *PI3K* mutations, indicating that therapies targeting these mutations may be effective [267,268]. Although currently there are no approved targeted therapies against *TP53* mutations, *TP53* mutations are correlated with elevated VEGF-A levels. Hence, *TP53* mutations may be used as biomarkers for predicting sensitivity to VEGF/VEGFR antagonists, such as bevacizumab in the clinic [218].

Metaplastic breast cancer has a high ribosomal protein L39 (RPL39) A14V mutation (97.5%) associated with poor overall patient survival. This is a gain-of-function mutation mediated by inducible nitric oxide synthase (iNOS). Although the function of RPL39 is unknown in breast cancer, N(G)-methyl-L-arginine acetate, a pan-iNOS inhibitor, decreased breast cancer cell proliferation in in vitro and in vivo mouse models [219]. This requires further validation using human samples. 

#### 9.2.2. miRNAs

Generally, the major miRNA therapies are oligonucleotide analogs and antagonists that inhibit the function of miRNAs. Similar to traditional gene therapy, miRNA analogs, also known as miRNA replacement therapy, are used to repair miRNAs with loss of function. Single-stranded oligonucleotides with complementary miRNA-sequences are used to silence the miRNA function of target proteins [269]. A recent study found that the expression of miR-206 is inversely correlated with VEGF expression in TNBC. Increased levels of miR-206 downregulated the expression of MAPK3, VEGF, and SOX9. Importantly, miR-206 mimics inhibited cell invasion and angiogenesis in TNBC, presenting a possible and efficient therapeutic strategy for TNBC [220]. 

#### 9.2.3. circRNA

As described above, certain circRNAs are involved in various steps of tumorigenesis, including proliferation, migration, invasion, apoptosis, and drug resistance, such as Foxo3 circular RNA [270]. Therefore, circRNAs represent a promising therapeutic target for breast cancer therapy. Currently, several technologies provide partial or complete removal of oncogenic circRNAs, such as siRNA-based therapy [271], anti-sense oligonucleotide therapy [272], and CRISPER/Cas system [273]. The roles of circRNAs in cancer stem cells (CSCs) have been explored using the circRNA profile in breast cancer stem cells (BCSCs) and RNA-sequencing, and circVRK1 was found to inhibit BCSC expansion and self-renewal capacity, indicating that circVRK1 might be a potential and effective target for BCSCs [274]. Treatment with 2,3,7,8-tetrachlorodibenzo-p-dioxin (TCDD) induces circ_0001098 overexpression via the miR-3942-3p/BARD1 axis, thereby suppressing tumor growth and metastasis [221]. The discovery of the TCDD-circRNA-miRNA-mRNA axis may provide a new direction for the development of therapeutic strategies for breast cancer.

#### 9.2.4. Poly (ADP-Ribose) Polymerase (PARP) in *BRCA1/*2 Mutations

Targeting poly (ADP-ribose) polymerase (PARP) in *BRCA* mutations provides an additional opportunity to treat these tumors in the advanced stage [19]. The PARP inhibitors olaparib and talazoparib were approved by the FDA in 2018 for locally advanced or metastatic HER2- breast cancer patients with the *BRCA 1/2* mutation. The approval was based on the results of the phase III OlympiAD and EMBRACA trials [222,223,275]. Both trials randomly assigned patients to receive the PARP inhibitor or chemotherapy and had primary endpoints of progression-free survival (PFS). Compared to chemotherapy, olaparib demonstrated an improved PFS at 7 months versus 4.2 months (HR 0.58; 95% CI 0.43–0.80, *p* < 0.001). Similarly, talazoparib resulted in better PFS than chemotherapy, at 8.6 months versus 5.6 months, respectively (HR 0.54; 95% CI 0.41–0.71, *p* < 0.001) [223]. However, resistance to PARP inhibitors is common, and multiple mechanisms are involved. Thus, the development of novel combination therapies is required [19].

Recently, a phase II study further evaluated the pathologic response of talazoparib evaluated for 6 months for operable breast cancer with germline *BRCA* mutation; the primary endpoint was residual cancer burden (RCB) [276]. This study showed a substantial RCB-0 (pCR) rate (53%) and a 63% RCB-0/I rate. This warrants a larger and ongoing neoadjuvant trial [277].

#### 9.2.5. PI3K/Anti-Apoptotic Kinase (Akt)/mTOR

PI3K/Akt/mTOR is a major pathway that participates in the regulation of cell survival and proliferation, and the essential genes of the PI3K/Akt pathway, *PIK3CA*, *AKT1*, *AKT2*, and *PTEN*, are often altered in breast cancer [278]. The PI3K pathway is hyperactivated in 70% of breast cancers and 20–40% have *PIK3CA* mutations [279]. *PIK3CA* is the third most mutated gene in basal-like breast cancer, followed by retinoblastoma (*RB*) and *BRCA1/2*. *AKT1* and *AKT2* mutations activate Akt signaling in HR+/luminal breast cancer [280]. Not surprisingly, the PI3K/Akt/mTOR pathway and different receptor tyrosine kinases (RTKs) are the major targets for treating breast cancer. 

There are a number of clinical trials for PI3K and Akt inhibitors; taselisib (GDC-0032), a PI3K inhibitor, was most dedicated to ER+ breast cancer. There is also a phase Ib/II trial for TNBC patients, but in combination with enzalutamide (MDV-3100), an AR antagonist. There is a phase II trial for capivasertib (AZD5363), an Akt inhibitor, dedicated to TNBC; there is a phase Ib trial for MK2206, an allosteric AKT inhibitor, dedicated to metastatic breast cancer, both in combination with paclitaxel. Bimiralisib (PQR309), a PI3K/mTOR inhibitor, has one phase Ib/II trial for TNBC. Gedatolisib (PF-05212384) and vistusertib (AZD2014) have received the most clinical trials targeting PI3K/mTOR and mTOR for TNBC [224]. Intriguingly, mTOR inhibitors, such as temsirolimus and everolimus, have been proven to have secondary effects on angiogenesis in metaplastic breast cancer, suggesting that combining bevacizumab and temsirolimus/everolimus might be worthwhile for clinical trials [231].

#### 9.2.6. CDK4/6

CDK4/6 regulates the cell cycle and is required for tumor initiation and progression. In breast cancer, this pathway is hyperactive, and its inhibition could activate the tumor suppressor Rb, leading to G1 cell cycle arrest [281]. CDK4/6 inhibitors, palbociclib [225], ribociclib [226,227], and abemaciclib [228] have been approved for ER+ and HER2– advanced breast cancer. They are also able to overcome or delay resistance to endocrine therapy [281]. Therefore, palbociclib, ribociclib, and abemaciclib are approved in combination with endocrine therapy for treating metastatic HR+ breast cancer [239,240,241]. In addition, inhibiting CDK4/6 could also promote cytotoxic T-cell-mediated immunity against tumor cells by stimulating type III interferon production, thus enhancing tumor antigen presentation [282]. 

#### 9.2.7. Other Proteins

Androgen receptor (AR), a steroid hormone receptor, is gaining attention as both a prognostic marker and a potential therapeutic target in breast cancer. Up to 90% of ER+ tumors, a moderate number of HER2+ tumors, and approximately 30% of TNBC express AR. AR expression seems to be correlated with improved outcomes in ER+ early-stage disease. However, there was either no effect or association with poorer survival in HER2+ breast cancer. In TNBC, despite the lower pCR to neoadjuvant chemotherapy, AR expression showed the highest overall survival rate [283,284]. AR-targeted therapies, such as AR agonists, AR antagonists, and PI3K inhibitors, have shown promising results in clinical trials, and combinations of AR-targeted therapies with other drugs have also been investigated to improve resistance to AR-targeted therapies. However, there are some controversial results that require further preclinical and clinical studies.

Some therapies target a broad spectrum of RTKs. These pan-RTK inhibitors target multiple RTKs to suppress cell proliferation, angiogenesis, and survival. Cabozantinib (XL184) targets ten molecules and is under phase II randomized discontinuation trial (RDT) for ER+, HER2–, and TNBC (metastatic breast cancers). The overall response rate (ORR) during the lead-in stage and disease control rate at week 12 were 13.6% and 46.7%, respectively, in patients with ER+ disease of clinical activity [229].

Furthermore, Janus kinase (JAK), non-RTKs, and signal transducers and activators of transcription (STAT), which are activated by JAK, are associated with cell proliferation and survival [224]. Some JAK inhibitors are in clinical trials, with ruxolitinib serving as an example. The combination of ruxolitinib with capecitabine showed a greater ORR than the placebo + capecitabine group (28.9% vs. 13.7%, *p* = 0.024) in HER2-advanced breast cancer [258]. Results were also analyzed in HER2+ and TNBC [224].

It was discovered that ribosomal protein (RP) L32 was overexpressed in human breast cancer tissues and cells. RPL32 knockdown suppressed the migration and invasion of breast cancer cells in vitro and in vivo in a mouse model. Therefore, this study revealed that RPL32 has a potential oncogenic role, indicating that it may be a potential target for molecular targeted therapy in breast cancer treatment [230]. However, this needs to be further validated using more human clinical samples.

### 9.3. Immunotherapies

Since breast cancer is not considered highly immunogenic, it has not been studied extensively in immunotherapy. However, emerging evidence demonstrates that immune cells and immune gene signatures play roles in the prognosis of breast cancer (Section 6.3). Therefore, the effectiveness of immunotherapies for the treatment of breast cancer has been studied extensively in recent years. 

#### 9.3.1. Immune Checkpoint Pathway Inhibition

PD-L1 is currently the most commonly used biomarker for stratifying patients for immunotherapies [285]. The level of PD-L1 expression varies widely in different cancer types, and its expression is relatively low (approximately 20–40%) in breast cancer compared to other cancers, such as thymic and nasopharyngeal cancers. However, higher PD-L1 expression (up to 60%) was observed in TNBC than in other breast cancer subtypes [286,287]. It has been demonstrated that PD-L1 expression is correlated with greater responses to anti-PD-1/PD-L1 therapies in breast cancer [288,289]. However, there is a large discrepancy in PD-L1 expression using IHC, which varies from 6–92.4% [290,291]. Thus, it should be noted that there are still many technical issues for PD-L1 to be solved, such as antibody selection, validated scoring system, the methods of evaluation, the cellular compartment to be examined, or the type of cells for PD-L1 status determination before PD-L1 can be a reliable biomarker used in clinics [292]. 

Ipilimumab, an anti-CTLA-4 monoclonal antibody, was the first immune checkpoint inhibitor approved by the FDA in March 2011 for the treatment of metastatic melanoma. Subsequently, several other immune checkpoint inhibitors, such as the CTLA-4 inhibitor, tremelimumab, and antibodies against PD-1, nivolumab, and pembrolizumab have been approved by the FDA for treating advanced solid tumors [232]. Several phase I clinical trials examining immune checkpoint inhibitors in advanced breast cancer are promising. The phase I JAVELIN study was conducted in patients with metastatic breast cancer who were treated with avelumab, an anti-PD-L1 antibody, and the results showed that the ORR was 5.2%, with a higher ORR in PD-L1+ tumors (16.7%) and TNBC (22.2%) compared to HER2+ and ER+ breast cancer (2.6%) [233]. In the phase Ib KEYNOTE-028 study, the ORR in patients with metastatic ER+HER2- breast cancer with PD-L1 expression, treated with pembrolizumab, an anti-PD-1 antibody, was 12% [234]. The large discrepancies in the results might be due to the largely different patient populations, different PD-L1 expression cut-off values, and different assays used. Despite these differences, TNBC is consistently associated with better outcomes compared with other subtypes when treated with immunotherapy, as TNBC has a higher PD-L1 expression. In addition, TNBC is correlated with higher TIL infiltration, which can promote an immune response [232]. 

There are a number of methods that can monitor the treatment responses during immunotherapy. The levels of autoantibodies against TAAs in the serum of breast cancer patients can be used to monitor treatment responses for immunotherapy. Interestingly, it was found that autoantibodies decreased significantly in combination therapies, especially radiation, chemotherapy, and hormonal therapy [293], suggesting that immunotherapy may be beneficial to these patients. Tumor mutation burden (TMB) is another emerging predictive biomarker, and increased TMB is associated with treatment response to immune checkpoint inhibitors in different cancer types [294]. However, the association between TMB and the clinical benefits of immune checkpoint inhibitors in breast cancer is not fully understood, and further studies are required to validate this. In addition, tumor immune microenvironment features, such as TILs and interferon-inflammatory immune gene signatures, are reported to be correlated with the treatment response to immune checkpoint inhibitors [285]. A recent study demonstrated that a tumor inflammation signature incorporating 18 genes that measure adaptive immune response within tumors is correlated with clinical sensitivity to PD-1/PD-L1 immunotherapy [295]. However, the variability resulting from this signature should be further improved by a better understanding of the immune status of untreated patients. It was also recently reported that effector CD8 T cells and their associated immune checkpoint molecules could be used to robustly predict treatment response to checkpoint inhibition. 

#### 9.3.2. T Cells

Several adoptive T cell therapy approaches, including collection, ex vivo expansion, and reinfusion of activated T cell lymphocytes, have been investigated in breast cancer patients. The results of a phase I immunotherapy trial evaluating anti-CD3 × anti-HER2 bispecific antibody (HER2Bi)-armed anti-CD3-activated T cells (ATC) in metastatic breast cancer showed that the OS was improved. Therefore, this therapeutic strategy utilizing HER2Bi-armed T cells for metastatic breast cancer was safe, and it is worthwhile to conduct phase II trials [236]. 

Emerging data have shown the role of TILs as a predictive biomarker for determining the response to chemotherapy in TNBC and HER2+ breast cancer. In the GeparSixto trial, increased levels of stromal TILs predicted pCR with the addition of carboplatin to an anthracycline-taxane combination [296]. Hence, it is important to identify effective biomarkers for predicting treatment responses to immunotherapies to maximize their benefits. 

#### 9.3.3. Chemokines

Chemokines have been proven to promote tumor cell proliferation, invasion, and migration, and regulate immune cell trafficking, indicating an important role in the TME. Therefore, immunotherapy targeting the chemokine superfamily network in the TME could be a potential therapeutic strategy for cancer progression and metastasis [196,297,298].

### 9.4. Combination Therapies

Considering the complicated interplay between different molecules during tumorigenesis and crosstalk between different signaling pathways, it is necessary to develop combination therapies. Many have been examined or evaluated in clinical trials. Indeed, in most cases, combination therapy is more effective than a single agent. 

#### 9.4.1. Combining Targeted Therapy and Endocrine Therapy

In a phase 1b study of alpelisib, an α-specific PI3K inhibitor, and fulvestrant in ER+ metastatic breast cancer with the *PIK3CA* mutation, promising results were recently declared [299,300]. The benefit of combining alpelisib and fulvestrant for HR+, HER2- advanced breast cancer with the *PIK3CA* mutation was further confirmed by the follow-up phase III SOLAR-1 trial. In the *PIK3CA* mutant cohort, when compared to fulvestrant with placebo, treatment with fulvestrant and alpelisib showed significantly extending PFS (11.0 months vs. 5.7 months) compared to fulvestrant with placebo [237]. 

In addition, in a phase II clinical trial, dovitinib, a pan-inhibitor of FGFR1-3, VEGFR, and PDGFR, combined with fulvestrant, increased PFS in postmenopausal women with HER2-, HR+ breast cancer [224]. 

#### 9.4.2. Combining Chemotherapy and Immunotherapy

Most subtypes of breast cancer are poorly immunogenic, which may result from insufficient antigen presentation for T cell activation. Chemotherapy and radiotherapy can overcome this problem by inducing specific antitumor immune responses by inducing immunogenic tumor cell death. Consequently, combination strategies of immunotherapy and chemotherapy/radiotherapy are being extensively evaluated in prospective clinical trials, mostly for immune checkpoint blockade [232]. 

Promising results have been observed in phase II, an adaptively randomized I-SPY2 trial combining pembrolizumab, an anti-PD-1 antibody, and taxane- and anthracycline-based neoadjuvant chemotherapies for high-risk stage II/III breast cancer. The final estimated pCR rates were 44% vs. 17%, 30% vs. 13%, and 60% vs. 22% for pembrolizumab vs. neoadjuvant chemotherapy control in ERBB2-, HR+/ERBB2-, and TNBC, respectively, suggesting that checkpoint inhibition in early-stage, high-risk, and ERBB2- breast cancer is highly likely to be successful in a phase III trial [242]. In the phase III KEYNOTE-522 trial for stage II or stage III TNBC, the pCR rates were 64.8% and 51.2% in the pembrolizumab plus chemotherapy group and the placebo with chemotherapy group, respectively, with a significant difference of 13.6% (95% CI 5.4–21.8, *p* < 0.001) [243]. Thus, the KEYNOTE 522 phase III trial further validated the results of the phase II I-SPY2 trial.

Additionally, the phase III IMpassion trial in previously untreated metastatic TNBC evaluated the combination of atezolizumab, an anti-PD-L1 antibody, and nab-paclitaxel. The results revealed no significant difference in overall survival between the treatment groups and the control group, but suggested a clinically meaningful overall survival benefit for the PD-L1+ subgroup [244,245]. Thus, nab-paclitaxel in combination with PD-L1 blockade atezolizumab for metastatic TNBC and at least 1% PD-L1 expression was approved by the FDA in March 2019 [244].

Alternatively, a phase II GeparNeuvo trial evaluated the combination of durvalumab, an anti-PD-L1 antibody with taxane-containing chemotherapy for early-stage TNBC [247]. The primary endpoint was pCR, which was 53.4% (95% CI 42.5–61.4) and 44% (95% CI 33.5–55.3) in the durvalumab and placebo groups respectively; this was not significantly different (*p* = 0.224). However, patients treated with a 2-week run-in of durvalumab received a clinical benefit in comparison with the placebo [277]. The studies mentioned above all highlighted the improved outcome of PD-1/PD-L1 blockade by chemotherapy.

For adoptive T cell therapy, a phase II study (NCT01147016) to evaluate the safety and efficacy of the combination of neoadjuvant chemotherapy and HER2Bi armed anti-CD3 activated T cells is being conducted [232]. 

#### 9.4.3. Combining Radiation Therapy and Immune Therapy

Numerous studies have reported the immunomodulatory effects of radiation therapy. The effect of radiotherapy induces an endogenous antigen-specific immune response and provides a rationale for combining radiation with checkpoint blockade in the clinic [251,301]. Prospective clinical trials, combining pembrolizumab and hypofractionated radiotherapy (HFRT) for metastatic cancers (NCT02303990) are evaluating the efficacy and safety of the combination treatment [232,302].

#### 9.4.4. Immunotherapy Targeting Multiple Checkpoint Molecules

In ER- tumors, multiple checkpoint molecules should be evaluated, as ER- tumors often co-express these molecules, which may make monotherapy ineffective. Indeed, the combination of durvalumab, a PD-L1 inhibitor, and tremelimumab, a CTLA-4 inhibitor, resulted in an increased ORR of 43%, approximately 2-fold in TNBC compared to the monotherapy [248]. An ongoing clinical trial (NCT02834013) evaluating the combination of anti-CTLA-4 (ipilimumab) and anti-PD-1 (nivolumab) blockade in rare tumor types including metaplastic breast cancer have been conducted [303].

#### 9.4.5. Combining Targeted Therapy and Immune Therapy

In solid tumors, resistance eventually develops in targeted therapies. In contrast, immunotherapy can induce durable responses. Immune evasion is one of the major obstacles to the efficacy of cancer immunotherapies, but epigenetic therapy may have immunomodulatory activities, leading to improved efficacy of cancer immunotherapies [304]. The potential use of combining epigenetic therapy and immunotherapy is currently being investigated in a phase II study of 5-azacitidine and entinostat in patients with metastatic HER2- breast cancer or TNBC (NCT02453620) [249]. However, the primary endpoints were not met. In a phase II study, the combination of vorinostat, tamoxifen, and pembrolizumab was evaluated in ER+ breast cancer (NCT02395627) [250]. 

It is proposed that targeting specific chemokine networks can influence the trafficking and infiltration of immune cells into tumors, as mentioned in Section 7.1. However, clinical trials of chemokine-targeted therapy have not yielded promising results in breast cancer with bone metastasis. This may be due to binding to multiple receptors by chemokine ligands, making the function of a single chemokine antagonist useless [297]. Hence, combination therapy targeting chemokine networks and other current therapies, such as immune therapies and traditional therapies including chemotherapy, radiotherapy, and endocrine therapy, could provide better clinical benefits in breast cancer patients with bone metastasis [196].

In addition, a phase II study evaluating PARP inhibitors, niraparib, in combination with immune checkpoint inhibitors, pembrolizumab, in advanced or metastatic TNBC has shown promising results [252]. Another trial (NCT01837602) evaluating engineered T cells combined with c-Met, expressed in ∼50% of breast tumors, was conducted for locally advanced and metastatic breast cancer [305]. The results showed that intratumoral injections of mRNA c-Met-CAR T cells were well tolerated and induced an inflammatory response within tumors.

#### 9.4.6. Combining Targeted Therapy and Chemotherapy

There are a number of clinical trials evaluating PARP inhibitors in the neoadjuvant setting. In the neoadjuvant I-SPY2 phase II trial in stage II/III breast cancer, veliparib, PARP inhibitor, and carboplatin with paclitaxel arm were compared with the paclitaxel alone arm, the estimated pCR rates were 51% and 26%, respectively, suggesting the clinical utility of the combination of veliparib with carboplatin and paclitaxel in a phase III clinical trial [254]. The phase III BrighTNess trial for stage 2 and 3 TNBC, received either veliparib + paclitaxel + carboplatin, paclitaxel + carboplatin + veliparib placebo, or paclitaxel + carboplatin placebo + veliparib placebo. The pCR rates were 53%, 58%, and 31% in the veliparib + carboplatin + paclitaxel arm, in the paclitaxel + carboplatin arm, and in the paclitaxel monotherapy arm, respectively [255]. These two results are consistent and indicate that the addition of carboplatin, but not veliparib, to standard neoadjuvant chemotherapy might benefit patients with early-stage high-risk TNBC. 

Most therapies target the PI3K/AKT/mTOR pathway. For targeting AKT, in the phase II PAKT trial for untreated metastatic TNBC, a combination of the AKT inhibitor capivasertib and paclitaxel resulted in significantly longer PFS and OS. The benefits were more pronounced in patients with PIK3CA/AKT1/PTEN-altered tumors [256]. The phase III CapiTello290 study (NCT 039997123) further evaluated capivasertib combined with paclitaxel for advanced TNBC [277]. Another AKT inhibitor, ipatasertib, was evaluated in the phase II LOTUS trial for untreated metastatic TNBC receiving either ipatasertib or placebo in combination with paclitaxel. Indeed, PFS was longer in patients who received ipatasertib than in those who received the placebo [257]. Phase III IPATunity130 is currently evaluating ipatasertib and paclitaxel for PIK3CA/AKT1/PTEN-altered advanced HER-2- breast cancer (NCT03337724) [277].

A phase I trial intended to target mTOR— and which involved metastatic metaplastic breast cancer treated with liposomal doxorubicin (D) and bevacizumab (A), with either temsirolimus (T) or everolimus (E) (DAT/DAE)—revealed that patients with advanced metaplastic breast cancer treated with mTOR-based systemic therapy had better long-term outcomes than those with nonmetaplastic TNBC, indicating that metaplastic histology may benefit from drugs targeting the PI3K/Akt/mTOR pathway [259]. Temsirolimus has also been studied in combination with bevacizumab and other chemotherapeutic agents, including platinum, taxanes, and anthracyclines. Comparing with non-metaplastic TNBCs, metastatic MPBC patients treated with DAT/DAE had better clinical outcomes, further supporting the treatment of this combination therapy for this particular subtype [259]. 

#### 9.4.7. Combining Immunotherapy and Endocrine Therapy

After exposure to trastuzumab, an increase in the signature of immune cell admixture (immune index) was associated with a higher complete pathological response rate in HER2+ breast cancer. Furthermore, CD4+ follicular helper T cells and PD-L1 were upregulated by trastuzumab [217]. These results suggest that the combination of trastuzumab and immune checkpoint inhibitors may be a novel strategy for HER2+ breast cancer, but this requires validation in large prospective studies.

## 10. Conclusions and Future Directions

With advancements in molecular technologies, such as comprehensive genomic profiling, microRNA expression profiling, and protein profiling, all types of biomarkers have been identified and intensively characterized for diagnosis, drug resistance, and prognosis for breast cancer. The development of cancer-specific autoantibodies may provide a complementary strategy to mammography for diagnosing breast cancer patients. Some biomarkers have been validated by clinical trials and thus should be clinically available, whereas some are promising biomarkers that need to be further validated by clinical trials. Further improvements are required before a novel biomarker can be applied as a standard diagnostic tool in the clinic, by incorporating standardized protocol and cutoff values, and thorough assessment of the sensitivity, specificity, reproducibility, stability, and safety of the assay. 

In the rapidly advancing field of personalized medicine, the ability of the promising biomarkers to screen and select certain subtypes of breast cancer to receive certain treatments to maximize treatment efficacy for breast cancer patients. A large number of novel targeted agents for breast cancer treatment have entered clinical validation. The emergence of immunotherapies offers breast cancer patients more opportunities to be cured. Improving responses to immunotherapy will require both improvements in T cell recruitment and inhibition of the strong immunosuppressive effect of TME. Many different combinations of strategies have been developed and are becoming increasingly complex. Combination therapy incorporating immunotherapy has great potential to overcome breast cancer resistance mechanisms. Alternatively, chemotherapy and radiation may potentially enhance the immune response when combined with immunotherapy.

In summary, it is important to note that the macromolecules and cells discovered as the promising diagnostic or prognostic biomarkers for breast cancer are mostly involved in tumor cell proliferation, invasion, migration, angiogenesis, survival, drug resistance, and immune response. Therefore, these biomarkers may also serve as the potential therapeutic targets for designing the therapeutic strategies or combination therapies to achieve personalized or precise treatments for breast cancer patients in the future.

## Figures and Tables

**Table 1:** Biomarkers discovered recently for breast cancer.

Type	Biomarkers	Clinical Value	Clinical Validation/Research Design	References
DNA	Immune response-related genes (*BTN3A2*, *CD2* and *TRBC1*)	may be used to identify patients with a good prognosis in HR−/HER2+ breast cancer.	Measured the tissues from 819 breast cancer patients.	[30]
	Immunity genes (*APOBEC3G*, *CCL5*, *CCR2*, *CD2*, *CD27*, *CD3D*, *CD52*, *CORO1A*, *CXCL9*, *GZMA*, *GZMK*, *HLA-DMA*, *IL2RG*, *LCK*, *PRKCB*, *PTPRC*, and *SH2D1A*)	immunity gene expression was an important parameter for prognosis.	Tested on 225 breast tumor FFPE tissues.	[31]
	T helper type-1 gene signatures (*IFNG*, *STAT1*, *GRZM*, *CXCL9*)	are correlated with favorable clinical outcome, particularly in ER- tumors.		[32,33,34]
	methylated *14-3-3 σ*	as a blood-based biomarker for breast cancer diagnosis.	meta-analysis	[35]
	methylated *APC* and *RARβ*_2_	might be valuable serum-based molecular markers for early detection of early-stage breast cancer, low grade tumors and TNBC.	Tested on serum samples from 121 breast cancer patients, 79 patients with benign breast diseases, and 66 healthy controls.	[36]
	*S100P* and *HYAL2* hypomethylation	as breast cancer biomarkers for early stage detection.	*S100P*: Validation I: 235 familial breast cancer cases and 206 controls; Validation II: 189 sporadic breast cancer cases and 189 controls; Validation III: 156 sporadic breast cancer cases and 151 controls. *HYAL2*: first validation round: 338 breast cancer cases and 507 controls; second validation round: 189 breast cancer cases and 189 controls.	[37,38]
	long noncoding RNA 299 gene (*LINC00299*) methylation	for early detection of TNBC in young women.	Examined blood samples of 154 TNBC cases and 159 breast cancer-free matched controls.	[39]
	*ESR1* mutations	1. *ESR1* Y537S mutation promotes resistance to fulvestrant. 2. may have clinical utility in directing further endocrine therapy. 3. *ESR1* mutations are prevalent in ER-positive aromatase inhibitor-treated metastatic breast cancer predicting its prognosis.	1. Testing the blood samples of 195 patients from the PALOMA-3 cohort; 2. In the SoFEA trial, plasma samples of 162 patients were tested; in the PALOMA3 trial, plasma samples of 360 patients were tested. 3. In the BOLERO-2 cohort, 541 plasma samples were examined.	[40,41,42]
	*TP53* mutation	associated with better prognosis in metaplastic breast cancer with increased RFS and OS.	Examined the clinical outcomes data of 52 archived samples.	[43]
	a 14-gene prognostic signature (*PFKL*, *P4HA2*, *GRHPR*, *SDC3*, *PPP1R15A*, *SIAH2*, *NDRG1*, *BTG1*, *TPD52*, *MAFF*, *ISG20*, *LALBA*, *ERRFI1*, and *VHL*)	could serve as a potential prognostic biomarker for breast cancer.	Clinical data from 1097 cases were obtained from the TCGA database. 113 adjacent normal samples and 1039 breast cancer patients were followed-up for ≥1 month.	[44]
	28-CpG based methylation panel	could independently predict the overall survival of breast cancer patients. Patients with high methylation risk were associated with tumor heterogeneity and poor survival.	The DNA methylation profile of The Cancer Genome Atlas Breast Invasive Carcinoma (TCGA-BRCA) included a total of 890 breast cancer samples. A total of 62, 118, 188, 70, and 58 breast cancer samples were included in GSE37754, GSE72245, GSE75067, GSE78754, and GSE72251. 40 normal breast samples and 80 breast cancer samples in GSE666952.	[45]
MicroRNAs	miR-21 and/or miR-221	can be successfully applied as breast cancer biomarkers.	Tested the sera of 50 patients with breast cancer, 25 fibroadenoma, and 25 healthy controls.	[46,47]
	six miRNA signature, miR-21, miR-221, miR-210, miR-195, miR-145, and let-7a	for early detection of TNBC.	Examined 85 paired tumor tissues and sera with an equal number of adjacent normal tissue margins and normal sera from healthy women and 15 benign fibroadenomas.	[48]
	miR-21	promotes the transformation and development of breast cancer.	Examined on blood samples of 30 female patients with breast tumors and 30 with benign breast lesions	[49]
	Exosomal miR-1246 and miR-21	for detection of breast cancer.	Tested the plasma of 16 patients with breast cancer and 16 healthy control subjects.	[50]
	five-miRNA signature, miR-1246, miR-1307-3p, miR-4634, miR-6861-5p, and miR-6876-5p	for detection of early stage breast cancer.	Tested 1280 serum samples of breast cancer patients, 2836 serum samples from non-cancer controls, 451 from patients with other types of cancers, and 63 from patients with non-breast benign diseases.	[51]
	The eight-marker signature (miR-16, let7d, miR-103, miR-107, miR-148a, let-7i, miR-19b, and miR-22)	for early detection of breast cancer including younger women.	Tested plasma from 127 sporadic breast cancer cases and 80 healthy controls.	[52]
	a 9-miRNA profile	for early detection of breast cancer.	Examined 116 blood samples including 36 with breast cancer.	[53]
	miR-1204	could be a novel prognostic/diagnostic biomarker for breast cancer patients.	Tested sera from 144 breast cancer patients and 38 healthy controls.	[54]
	combination of miR-181b-5p, miR-200b-3p, miR-200c-3p, and miR-203a-3p	could be potential diagnostic biomarkers for inflammatory breast cancer.	Examined tissue specimens of 18 non- inflammatory breast cancer and 17 inflammatory breast cancer patients.	[55]
	miR-140 and miR-196a	both miR-140 and miR-196a are promising biomarkers for the diagnosis of breast cancer.	Tested 110 cases of breast cancer and their adjacent non-tumor tissues.	[56]
	miR-26a/26b	may be useful markers of the progression of breast cancer.	Examined 29 pairs of fresh breast cancer and adjacent tissues.	[57]
	miR-26b	inhibited TNBC cell proliferation and tumor growth.	-	[58]
	miR-182	contributed to cell progression.	45 patients with breast cancer.	[59]
	miR-224	inhibited proliferation and migration of breast cancer cells.	Examined serum samples from 45 patients with breast cancer.	[60]
	miR-124-3p	reduced breast cancer cell proliferation and metastasis.	Tested 30 breast cancer and normal breast tissues.	[61]
	miRNA-17 and miRNA-20b	resistance to taxol in breast cancer patients increased with the loss of miRNA-17 and miRNA-20b.	55 pairs of breast cancer tissues and adjacent normal tissues were examined.	[62]
	miR-18a	overexpression directly led to Dicer repression and confers paclitaxel resistance in TNBC.	Tested 20 TNBC patient tissues.	[63]
	miR-90b, 130a, 200b, and 452	contribute to chemoresistance.	-	[64]
	miRNAs 221 and 222	chemoresistance to fulvestrant, doxorubicin, or trastuzumab.	-	[64]
	miRNA 320a	chemoresistance to paclitaxel.	-	[64]
	miRNAs let-7, 181a and 145	chemoresistance to doxorubicin, tamoxifen, or epirubicin.	-	[64]
	miRNA 125b	chemoresistance to tamoxifen, letrozole, anastrazole or fulvestrant.	-	[64]
	miR200c and miR489	downregulation of miR200c and miR489 were correlated with better prognosis.	-	[64]
	miR484 and miR4443	upregulation of miR484 and miR4443 were associated with better prognosis.	-	[64]
	miR520h and miR125b	upregulation of miR520h and miR125b were correlated with poor prognosis.	-	[64]
	miR125b and miR21	could be novel, noninvasive predictive markers for neoadjuvant chemotherapy response and prognosis in breast cancer.	Examined 118 stage II/III breast cancer patients and 30 healthy adult women.	[65]
	miR-106b	is a putative plasma marker for risk assessment in patients with breast cancer.	Examined the tissue and plasma samples from 173 patients with primary breast cancer and 50 women with fibroadenoma.	[66]
	pre-miR-488	could be a novel prognostic biomarker for predicting recurrence in breast cancer patients.	Tested the blood from 356 female patients with breast cancer without distant metastases, preoperative therapy or previous treatment for various cancers, 330 invasive ductal carcinomas (IDC), 26 were ductal carcinomas in situ (DCIS), and 11 healthy volunteers.	[67]
	miR-130b	contributes to MDR through PI3K/Akt signaling pathway.	Tested 29 pairs of breast cancer tissues and their adjacent noncancerous tissues.	[68]
	miR-9	inhibit metastasis.	-	[69]
	miR-205	inhibit metastasis.	Tested on 40 pairs of TNBC and their adjacent normal breast tissues.	[70,71]
	miR-628	inhibit metastasis.	-	[72]
cicRNAs	hsa_circ_0001785	the potential diagnostic biomarker for breast cancer.	Examined the plasma of 57 breast cancer patients and 17 age-matched healthy individuals.	[73]
	Combination of hsa_circ_006054, hsa_circ_100219, and hsa_circ_406697	may be diagnostic biomarker for breast cancer.	Tested 51 breast cancer and adjacent normal tissues.	[74]
	hsa_circ_0001982	hsa_circ_0001982 knockdown suppressed breast cancer cell proliferation and invasion and induced apoptosis by targeting miR-143.	Examined 29 breast cancer tissues and adjacent normal tissues.	[75]
	circRNA-000911	enhanced expression of circRNA-000911 suppressed cell proliferation, migration and invasion, and promoted the apoptosis of breast cancer cells.	Human circRNA microarray analysis.	[76]
	circ-ABCB10	circ-ABCB10 knockdown suppressed the proliferation and increased apoptosis of breast cancer cells.	Tested 36 cancer and adjacent noncancerous tissues.	[77]
	circGFRA1	Knockdown of circGFRA1 inhibited proliferation and promoted apoptosis in TNBC.	Examined 51 TNBC tissues and their paired adjacent normal tissues.	[78]
	circ_0006528	may play a role in breast cancer chemoresistance.	-	[79]
Protein	4-test combination of TAP + CEA + CA125 + CA15-3	higher sensitivity than the traditional test, i.e., CEA, CA125, or CA15-3 and may be auxiliary used in early screening.	Tested on blood of 261 women with operable benign breast disease and 348 with breast cancer.	[80]
	TFF1, TFF2 and TFF3	for breast cancer screening.	Examined sera in 94 breast cancer patients and 84 health check females, and breast cancer tissues.	[81]
	Pleiotrophin (PTN)	PTN could be a potential biomarker for the presence of breast cancer.	Tested sera in 105 breast cancer patients and 40 healthy volunteers using ELISA. In addition, PTN expression was examined in 80 BC tissues in a nested case-control study by immunohistochemistry.	[82]
	Combination of miR-127-3p and HE4	Greatly improved the sensitivity of breast cancer diagnosis and may be a candidate biomarker for early detection and diagnosis of breast cancer.	Examined plasma in 102 patients with breast cancer, and 87 patients with benign breast tumors and 90 healthy volunteers as control.	[83]
	Combination of VEGF and CA 15-3	showed the highest usefulness in the diagnosis of early breast cancer.	Tested plasma in 100 breast cancer patients, and 50 patients with benign breast tumors, and 50 healthy women as control.	[84]
	Combination of AGR3 and AGR2	showed the potential usability of AGR3 and AGR2 as biomarkers for blood-based early detection of human breast cancer.	Examined 190 breast carcinomas and 39 normal breast tissues; 40 breast cancer and 40 healthy serum samples.	[85]
	COL11A1, COMP, and COL10A1	may be useful in diagnostic assessment for breast cancers	Discovery dataset: 50 healthy donors, 42 patients with benign breast disease, and 52 patients with invasive breast cancer; validation cohort: 52 healthy donors, 49 benign breast disease, and 66 invasive breast cancer.	[86]
	CA15-3 included in the diagnostic panel constituted of 4 protein peaks [m/z 3972, 6850, 8115 (Bc2), and 8949 (Bc3)]	distinguished invasive ductal carcinoma from healthy controls and benign breast diseases.	Tested the sera from 62 patients with invasive ductal carcinoma, and 47 non-cancerous individuals (16 healthy controls and 31 patients with benign breast diseases).	[87]
	Serum autoantigens (LGALS3, PHB2, MUC1 and GK2) in combination with CA 15-3	had better diagnostic values compared with anti-CA 15-3 alone for early-stage breast cancer.	Examined the sera from 100 breast cancer patients and 50 healthy subjects.	[88]
	A combination of six antigens, RAD50, PARD3, SPP1, NY-BR-62, and NY-CO-58	could discriminate breast cancer patients from healthy controls.	Tested the sera of 112 patients with breast cancer and 35 patients with no neoplasm (control group); Cancer and non-cancerous breast tissue samples were obtained from 17 female patients with primary breast carcinomas and 7 patients with fibrocystic disease.	[89]
	A combination of serum protein biomarkers and tumor associated autoantibodies	the benefit of the integration of SPB and TAAb for detecting breast cancer.	Using a retrospective cohort of sera from 18 participants with no breast diseases, 92 participants with benign breast diseases, and 100 participants with breast cancers.	[90]
	Sex hormones: estradiol, testosterone, and SHBG	Integration of hormone measurements in clinical risk prediction models may represent a strategy to improve breast cancer risk stratification.	Tested blood of 1217 breast cancer cases (430 pre- and 787 postmenopausal) and 1976 matched Controls.	[91]
	CCL25/CCR9 chemokine signaling	promotes migration and invasion in different cell lines by selective regulating several EMT markers.	-	[92]
	CCL18-PITPNM3 chemokine signaling	promotes the invasion and metastasis of breast cancer through the PI3K/Akt/GSK3β/Snail pathway.	-	[93,94]
	TNBC-specific RBP, NONO	NONO is highly expressed in TNBC and is associated with poor patient outcomes, a potential therapeutic target in TNBC.	Tested on tissue microarray.	[95]
	Peptides KNG1_K438-R457_ and C3f_S1304-R1320_	differentiate *BRCA1* mutant breast cancer from sporadic B breast cancer and cancer-free *BRCA1* mutant carriers.	Examined on serum samples from 55 carriers of hereditary *BRCA1* mutations, of whom 28 were diagnosed with breast cancer, and 27 remained cancer-free, 39 were diagnosed with sporadic breast cancer, and 38 were healthy controls.	[96]
Lipid	27-hydroxycholesterol	may offer a novel breast cancer risk strategy.	Tested on sera of 530 incident invasive breast cancer cases and 1036 control participants from Heidelberg cohort of EPIC.	[97,98]
Exosome	fibronectin	This liquid biopsy to detect fibronectin on circulating extracellular vesicles could be a promising method to detect early breast cancer.	Tested on plasma samples from 70 disease-free individuals, 240 breast cancer patients, 40 breast cancer patients after surgical resection, 55 patients with benign breast tumor, and 80 patients with non-cancerous diseases (thyroiditis, gastritis, hepatitis B, and rheumatoid arthritis.	[99]
	Del-1	is a promising marker for identification of patients with early-stage breast cancer and distinguish breast cancer from benign breast tumors and noncancerous diseases.	Measured in plasma samples from 81 healthy controls, 269 patients with breast cancer, 50 breast cancer patients after surgical resection, 64 patients with benign breast tumors, and 98 patients with noncancerous diseases.	[100]

**Table 2:** Immune cells and other non-cancer cells as the biomarkers for breast cancer.

Cell Types	Prognosis/Treatment	References
T cells (Tregs)	better prognosis in lymph node negative, primary breast cancer patients including those with stages I–III.	[32,33,34,101,102,103]
CD8 T cells	were predictive for response to checkpoint inhibitors.	[104]
B cells	1. better prognosis in lymph node negative, primary breast cancer patients including those with stages I–III, ER- breast cancer, highly proliferating luminal B breast cancer, and 2. improved outcome in HR+ breast cancer.	[101,102,105,106]
Plasma cells	better prognosis in ER- breast cancer and highly proliferating luminal B breast cancer.	[106]
TILs	1. The frequency of TILs is usually high in the more aggressive breast cancer subtypes. TIL frequency was found to be a superior prognostic marker; 2. were predictive for response to checkpoint inhibitors, 3. was associated with improved responses to trastuzumab or lapatinib in HER2+ breast cancer.	[33,104,106,107,108]
Macrophages	associate with survival in basal-like breast cancer.	[103,108,109,110]
MDSCs	are correlated with poor survival in ER- tumors.	[109,110]
Neutrophils	1. are associated with poor breast cancer survival; 2. inhibiting leukotriene-generating enzyme arachidonate 5-lipoxygenase (Alox5) abrogates neutrophil pro-metastatic activity and consequently reduces metastasis.	[108,111]
NK cells	were found significantly depleted from peripheral blood compared to pretreatment levels after chemotherapy.	[102]
myeloid dendritic cell	improved outcome in HR+ breast cancer.	[105]
astrocytes	may provide new opportunities for effective anti-metastasis therapies, especially for brain metastasis patients.	[112]

**Table 3:** The therapeutic implications of breast cancer cell biomarkers.

Therapeutic Strategies	Targets	Effects/Indications	Treatment	References
Chemotherapy	Ki-67	ER-positive tumors with an elevated Ki-67	adjuvant docetaxel chemotherapy	[203,204]
Endocrine therapy	ER	For ER+ breast cancer	tamoxifen, fulvestrant, aromatase inhibitors	[205,206,207,208]
	HER2	For HER2+ breast cancer	trastuzumab, pertuzumab, Ado-trastuzumab emtansine, lapatinib and neratinib	[209,210,211]
	Ki-67	for hormone receptor positive breast cancer	neoadjuvant endocrine therapy	[212,213,214]
	TILs	for HR+ post-menopausal women	exemestane, tamoxifen	[215]
	gene expression of the T-cell marker, PD-1	For HR+ tumor	trastuzumab	[216,217]
Targeted therapy	*ESR1* mutation	for patients with metastatic breast cancer harboring the *ESR1* mutations	lasofoxifene, a selective estrogen receptor modulator	[19]
	*TP53* mutation	for predicting sensitivity to VEGF/VEGFR inhibitors in the clinic	VEGF/VEGFR inhibitors	[218]
	RPL39 A14V mutation	decreased in vitro proliferation and migration and in vivo tumor growth in mouse models	pan-NOS inhibitor N(G)-methyl-L-arginine acetate	[219]
	miR-206	inhibited TNBC cell invasion and angiogenesis	The miR-206 mimics	[220]
	circRNA_BARD1	induces the overexpression of circRNA_BARD1 and suppressed breast cancer tumorigenesis via miR-3942-3p/BARD1 axis.	2,3,7,8-tetrachlorodibenzo-p-dioxin (TCDD)	[221]
	Poly (ADP-ribose) polymerase (PARP) in *BRCA1/2* mutations	for germline *BRCA*-mutant metastatic breast cancer	Olaparib and talazoparib	[222,223]
	PI3K	for ER+ breast cancer	taselisib (GDC-0032)	[224]
	mTOR	for TNBC	vistusertib (AZD2014)	[224]
	PI3K/mTOR	for TNBC	bimiralisib (PQR309), gedatolisib (PF-05212384),	[224]
	CDK4/6	for ER+ and HER2− advanced breast cancer	palbociclib, ribociclib, and abemaciclib	[225,226,227,228]
	Receptor tyrosine kinase	for ER+, HER2– and TNBC patients	Cabozantinib, pan-RTK inhibitor	[229]
	ribosomal protein L32 (RPL32)	was upregulated in human breast cancer tissues and cells. It may be a novel target for molecular targeted therapy in breast cancer patients. Tested on the commercial microarray of breast cancer tissue containing 128 samples of infiltrating ductal carcinoma and six samples of infiltrating ductal carcinoma with infiltrating lobular carcinoma, in vitro breast cancer cell lines and in vivo mouse model.	-	[230]
	mTOR	for metaplastic breast cancer	Combination of temsirolimus/everolimus and bevacizumab	[231]
Immunotherapy	PD-1 and PD-L1	for PD-L1 positive breast cancer and TNBC	Anti-PD-1 antibody (pembrolizumab) and anti-PD-L1 antibodies (atezolizumab, avelumab, durvalumab)	[232,233,234,235]
	T cells	for patients with metastatic breast cancer	anti-HER2Bi armed antiCD3 activated T cells	[236]
	chemokines	inhibit cancer metastasis		[196]
Combination therapies	Ki-67	early decrease in Ki-67 at 3-week biopsy was associated with an increased likelihood of pathologic complete response in ER-positive, HER2-positive patients	neoadjuvant endocrine therapy combined with either T-DM1 or trastuzumab	[214]
	*PIK3CA* mutant	improved progression free survival in HR+HER2- advanced breast cancer with *PIK3CA* mutant	fulvestrant (selective estrogen receptor degrader) and alpelisib (PI3K inhibitor)	[237,238]
	FGFR1-3, VEGFR, and PDGFR	for postmenopausal patients with HER2-, HR+ breast cancer	Dovitinib (a pan-inhibitor of FGFR1-3, VEGFR, and PDGFR) and fulvestrant	[224]
	PI3K and AR	for TNBC	taselisib + enzalutamide (MDV-3100)	[224]
	Akt	1. for TNBC; 2. for metastatic breast cancer	1. capivasertib (AZD5363) + paclitaxel; 2. MK2206 + paclitaxel	[224]
	CDK4/6	for metastatic hormone-receptor positive breast cancer	combination of palbociclib, ribociclib, and abemaciclib (CDK4/6 inhibitors) and endocrine therapy	[239,240,241]
	PD-1	for the ERBB2-, HR+/ERBB2-, and TNBC	paclitaxel (chemotherapy) and PD-1 blockade (pembrolizumab)	[242,243]
	PD-L1	for metastatic TNBC and PD-L1 expression on immune cells (IC) occupying at least 1% of the tumor area	nab-paclitaxel (chemotherapy) and PD-L1 blockade (atezolizumab)	[244,245,246]
	PD-L1	for early stage TNBC	durvalumab, an anti-PD-L1 antibody and taxane-containing chemotherapy	[247]
	PD-L1 and CTLA-4	- durvalumab blocks the interaction of PD-L1 with PD-1 CD279; - for TNBC	durvalumab (anti-PD-L1 antibody) and tremelimumab (CTLA-4 inhibitor)	[248]
	DNA methyltransferase 1 (DNMT-1) and benzamide histone deacetylase inhibitor	for metastatic HER2- breast cancer or TNBC	5-azacitidine, DNMT-1 inhibitor, and entinostat, benzamide histone deacetylase inhibitor	[249]
	histone deacetylases (HDAC) and PD-1	ER+ breast cancer	vorinostat, HDAC inhibitor, tamoxifen, and pembrolizumab	[250]
	PD-1	stereotactic XRT induced endogenous antigen-specific immune responses	stereotactic radiotherapy (XRT) combined with anti-PD-1 checkpoint blockade immunotherapy	[251]
	PARP and PD-1	for advanced or metastatic TNBC	Niraparib, PARP inhibitor, and pembrolizumab, immune checkpoint inhibitors	[252]
	CXCR4	for metastatic breast cancer	CXCR4 antagonist POL5551 (PEM) with eribulin (a chemotherapeutic microtubule inhibitor)	[253]
	PARP	for stage II or III breast cancer	veliparib with carboplatin and paclitaxel	[254,255]
	AKT	for metastatic TNBC	AKT inhibitor capivasertib and paclitaxel	[256]
	AKT	for metastatic TNBC	AKT inhibitor ipatasertib and paclitaxel	[257]
	JAK1/2	for metastatic HER2− breast cancer	ruxolitinib and capecitabine	[258]
	mTOR, VEGF-A	for metastatic metaplastic breast cancer	1. liposomal doxorubicin, bevacizumab (VEGF-A inhibitor), with either temsirolimus or everolimus (mTOR inhibitors); 2. Temsirolimus in combination with bevacizumab and other chemotherapy agents including platinums, taxanes and anthracyclines.	[259]

## Abbreviations

27-HC	27-hydroxycholesterol
AGR	anterior gradient
Akt	anti-apoptotic kinase
APC	*adenomatous polyposis coli* and
C3	complement C3
CA15-3	carbohydrate antigen 15-3
CA125	cancer antigen 125
CEA	carcinoembryonic antigen
cfDNA	cell-free DNA
COMP	collagen oligomeric matrix protein
*CPXM1*	carboxypeptidase X
CRP	C-reactive protein
*DACH1*	Dachshund homolog 1
DCIS	ductal carcinomas in situ
DFS	disease-free survival
ECM	extracellular matrix
ELISA	enzyme-linked immunosorbent assay
EMT	epithelial-mesenchymal transition
ER	estrogen receptor
EPIC	European Investigation into Cancer and Nutrition
FAK	focal adhesion kinase
HR	hormone receptor
HER2	human epidermal growth factor receptor 2
*HOXA10*	Homeobox protein Hox-A10
iNOS	inducible nitric oxide synthase
IDC	invasive ductal carcinomas
IHC	immunohistochemistry
JAK	Janus kinase
KNG1	kininogen-1
ncRNAs	non-coding RNAs
miRNAs or miR	microRNAs
mTOR	mammalian target of rapamycin
NCOA3	nuclear receptor co-activator 3
NK cell	natural killer cell
ORR	overall response rate
OS	overall survival
PALOMA3	Palbociclib Combined With Fulvestrant in Hormone Receptor-Positive HER2-Negative Metastatic Breast Cancer After Endocrine Failure
pCR	pathologic complete response
PCR	polymerase chain reaction
PD-1	programmed cell death protein 1
PD-L1	programmed death-ligand 1
PTEN	phosphatase and tensin homolog
PTN	pleiotrophin
PI3K	phosphoinositide-3-kinase
PFS	progression-free survival
PR	progesterone receptor
*RASGRF1*	Ras-specific guanine nucleotide-releasing factor 1
*RAR-β2*	retinoic acid receptors-β2
RFS	recurrence free survival
Rb	retinoblastoma
SHBG	sex hormone–binding globulin
SoFEA	Study of Faslodex Versus Exemestane With or Without Arimidex
SOS1	SOS Ras/Rac guanine nucleotide exchange factor 1
STAT	signal transducers and activators of transcription
TAAs	tumor-associated antigens
TAMs	tumor-associated macrophages
TFF	trefoil factor
Th cell	T helper cell
TIL	tumor-infiltrating lymphocytes
TMB	tumor mutation burden
TME	tumor microenvironment
TNBC	triple-negative breast cancer
Tregs	regulatory T cells
VEGF	vascular endothelial growth factor
